# Transcriptional regulation of bacterial virulence gene expression by molecular oxygen and nitric oxide

**DOI:** 10.4161/viru.27794

**Published:** 2014-10-31

**Authors:** Jeffrey Green, Matthew D Rolfe, Laura J Smith

**Affiliations:** Krebs Institute; Molecular Biology & Biotechnology; University of Sheffield; Western Bank, Sheffield, UK

**Keywords:** FNR, iron–sulfur cluster, *Mycobacterium tuberculosis*, nitric oxide sensors, oxygen sensors, *Staphylococcus aureus*, WhiB-like proteins

## Abstract

Molecular oxygen (O_2_) and nitric oxide (NO) are diatomic gases that play major roles in infection. The host innate immune system generates reactive oxygen species and NO as bacteriocidal agents and both require O_2_ for their production. Furthermore, the ability to adapt to changes in O_2_ availability is crucial for many bacterial pathogens, as many niches within a host are hypoxic. Pathogenic bacteria have evolved transcriptional regulatory systems that perceive these gases and respond by reprogramming gene expression. Direct sensors possess iron-containing co-factors (iron–sulfur clusters, mononuclear iron, heme) or reactive cysteine thiols that react with O_2_ and/or NO. Indirect sensors perceive the physiological effects of O_2_ starvation. Thus, O_2_ and NO act as environmental cues that trigger the coordinated expression of virulence genes and metabolic adaptations necessary for survival within a host. Here, the mechanisms of signal perception by key O_2_- and NO-responsive bacterial transcription factors and the effects on virulence gene expression are reviewed, followed by consideration of these aspects of gene regulation in two major pathogens, *Staphylococcus aureu*s and *Mycobacterium tuberculosis*.

## Abbreviations

AIP: autoinducer peptide
Arc: Aerobic respiratory control
FNR: fumarate nitrate reduction regulator
GAF: cGMP-specific phosphodiesterase-adenylyl cyclase-FhlA domain
iNOS: inducible nitric oxide synthase
Isc: iron–sulfur cluster biosynthesis machinery
NOX: NADPH oxidase
PAS: Per-Amt-Sim domain
RNS: reactive nitrogen species
ROS: reactive oxygen species
TB: tuberculosis

## Introduction

**Figure 1.**
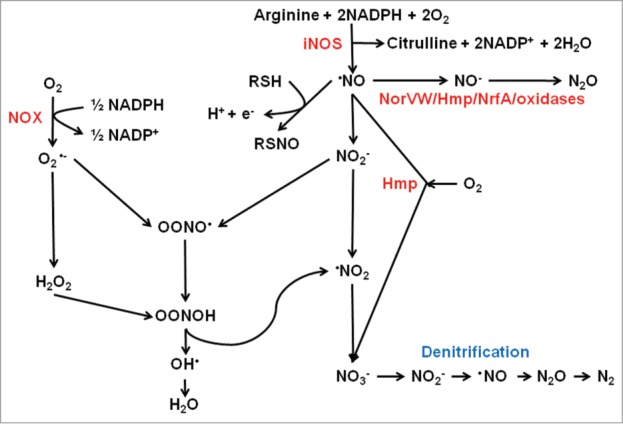
Interplay between reactive oxygen and reactive nitrogen species. The host enzyme NADPH oxidase (NOX) generates superoxide (O_2_^•−^) from O_2_. Aerobic metabolism within the pathogen inevitably results in side reactions in which successive one electron reductions of O_2_ yields the reactive oxygen species, O_2_^•−^, hydrogen peroxide (H_2_O_2_) and the hydroxyl radical (^•^OH). Nitric oxide (NO) is generated by the action of host inducible nitric oxide synthase (iNOS) (and by some bacteria that possess nitric oxide synthase). Nitric oxide is a reactive free radical and is a source of reactive nitrogen species such as nitroxyl (NO^−^), nitrosonium (NO^+^), and peroxynitrite (ONOO^−^), which is formed by reaction of NO with O_2_^•−^, or NO^−^ and O_2_, and peroxynitrous acid (OONOH). Nitric oxide reacts with thiol groups to modify activity by the formation of *S*-nitrosylated proteins (RSNO). Nitric oxide can be detoxified by the flavohemoglobin Hmp by conversion to nitrate (NO_3_^−^) in the presence of O_2_. Some bacteria are capable of denitrification in which NO_3_^−^ is converted to nitrogen gas (N_2_) via NO as an intermediate. In the absence of O_2_, the major detoxification mechanism in *E. coli* is the anaerobic NO reductase NorVW (O_2_-sensitive flavorubredoxin) that converts 2NO to N_2_O (nitrous oxide) and water. A similar reaction can be catalyzed by Hmp and NrfA in the absence of O_2_. Some terminal oxidases can also reduce NO to N_2_O, or by reaction of ferryl heme (Fe^4^^+^ = O^2−^) with NO generate NO_2_^−^.

Molecular oxygen (O_2_) and nitric oxide (NO) are freely diffusible diatomic gases because they are soluble in aqueous media but can partition into and cross biological membranes. Both gases have complex chemistries and play major roles in the host response to infection (**Fig. 1**). The NADPH oxidase (NOX) of professional phagocytes (e.g., macrophages and neutrophils) generates an “oxidative burst” by catalyzing the one electron reduction of O_2_ to superoxide (O_2_^•−^). A further one electron reduction of O_2_^−^ yields hydrogen peroxide (H_2_O_2_), one of the products resulting from the action of superoxide dismutase (2O_2_^−^ + 2H^+^ → H_2_O_2_ + O_2_). In the presence of ferrous ions (Fe^2+^) H_2_O_2_ undergoes Fenton chemistry to produce the hydroxyl radical (OH^•^).^1,2^ Superoxide, H_2_O_2_, and OH^•^ are collectively termed reactive oxygen species (ROS) and are capable of damaging many cell components, including DNA, proteins and membranes, resulting in bacterial death or bacteriostasis.^1,2^ The inducible nitric oxide synthase (iNOS), found in professional phagocytes, catalyzes the formation of NO from l-arginine and O_2_.^3^ Nitric oxide is a reactive lipophilic radical, which reacts with metalloproteins and protein thiols. Furthermore like O_2_^•−^, NO production leads to the formation of other toxic molecules collectively termed reactive nitrogen species (RNS). The most important RNS are nitroxyl (NO^−^), nitrosonium (NO^+^) and peroxynitrite (ONOO^−^); the last being formed as a result of the reaction of NO with O_2_^•−^, or from NO^−^ and O_2_.^4^ These RNS modify metal cofactors, protein cysteine, methionine, and tyrosine residues, with consequent bacteriostatic and bacteriocidal effects. Not surprisingly, bacterial pathogens have evolved mechanisms to sense O_2_ and NO and respond by deploying defensive mechanisms that detoxify ROS and RNS and repair oxidative and nitrosative damage to cell components. This review summarizes our current understanding of the mechanisms of O_2_ and NO perception by transcription factors and by examination of selected examples illustrates how bacterial cells use this information to control virulence gene expression and host–pathogen interactions.

## FNR, the Paradigm Of O_2_-Responsive Transcription Factors

**Figure 2.**
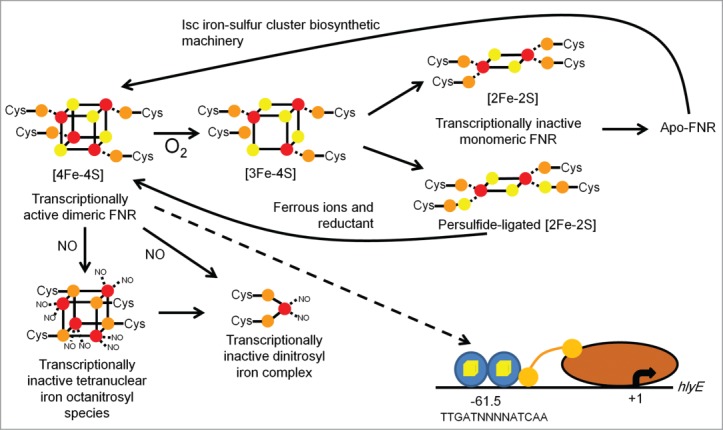
Scheme summarizing the changes in the FNR iron–sulfur cluster that occur upon reaction with O_2_ or NO and regulation of the *hlyE* gene. In *E. coli*, newly translated apo-FNR acquires a cubic [4Fe-4S] cluster (iron in red, cluster sulfur in yellow, Cys sulfur in orange) via the action of the iron sulfur cluster biosynthetic machinery (Isc). In the absence of O_2_, the [4Fe-4S] form of FNR is stable and cluster acquisition promotes dimerization and enhanced site-specific (consensus sequence: TTGATNNNNA TCAA) DNA-binding at target promoters, such as that encoding the cytolysin HlyE. Expression of *hlyE* is driven from a class I FNR-dependent promoter (FNR binding site located at −61.5 relative to the transcript start, +1) via interactions between the downstream subunit of FNR (blue oval with yellow cube) and the C-terminal domain of the α-subunit of RNA polymerase (brown). In the presence of oxygen (O_2_) the [4Fe-4S] cluster is converted to a planar [2Fe-2S] cluster via a [3Fe-4S] intermediate. This is accompanied by conversion of FNR from the DNA-binding competent dimeric form to the transcriptionally inactive monomer. During this process, cluster sulfide can be retained in the form of a persulfide-ligated [2Fe-2S] form of FNR, allowing facile repair of the cluster and a return to the [4Fe-4S] form. Prolonged exposure to O_2_ results in the breakdown of the [2Fe-2S] forms of the protein resulting in apo-FNR, which can acquire a [4Fe-4S] cluster by interaction with Isc. The FNR [4Fe-4S] cluster also reacts with NO yielding an octanitrosylated form and dinitrosyl iron complexes. Like O_2_, reaction with NO results in FNR inactivation.

Several well-known bacterial pathogens (e.g., *Escherichia coli*, *Klebsiella pneumoniae*, *Salmonella* Typhi, *Shigella dysenteriae*, and *Yersina pestis*) are facultative anaerobes capable of aerobic respiration, anaerobic respiration and fermentation. Thus, the ability to sense and respond to changes in O_2_ availability is essential for the competiveness of these bacteria. Both direct and indirect O_2_-sensing regulatory systems have been characterized in these bacteria, with the **F**umarate **N**itrate **R**eduction regulator (FNR) protein of the model bacterium *E. coli* K-12 being the paradigm of a direct O_2_-responsive transcription factor (**Fig. 2**).^5-7^

FNR is a member of the cyclic-AMP receptor protein family of transcription regulators. Under anaerobic conditions, FNR is activated by incorporation of an iron–sulfur cluster ([4Fe-4S]) coordinated by four essential cysteine residues (Cys-20, 23, 29, and 122), located within the N-terminal sensory domain of the protein.^8,9^ Iron–sulfur clusters are widespread, redox-active, biological structures composed of iron and sulfide that are most commonly held in proteins by four cysteine residue thiolates that act as coordinating ligands.^5-7^ The [4Fe-4S]^2^^+^ cluster acquired by FNR is one of the most common forms of iron–sulfur cluster. The [4Fe-4S]^2^^+^ cluster is a cube made up of two interpenetrating tetrahedra of iron (two Fe^3^^+^ and two Fe^2+^) and sulfide ions held by the essential cysteine residues of FNR interacting with the iron atoms at the vertices of the cube. The second common form of iron–sulfur cluster is the planar [2Fe-2S]^2+^ cluster, consisting of a [Fe_2_-(μ_2_-S)_2_] rhomb (rhombus) of two Fe^3+^ and two sulfide ions (both sulfide ions bridge two iron atoms hence the μ_2_-S designation), again most often coordinated by four cysteine residues. The [4Fe-4S]^2+^ and [2Fe-2S]^2+^ clusters can be inter-converted, sometimes via a [3Fe-4S]^1+^ intermediate. Inter-conversion of the cubic [4Fe-4S] and planar [2Fe-2S] clusters drives protein conformational changes that are mediated by the need to re-orientate the ligating cysteine residues to accommodate the change in the geometry of the iron–sulfur cluster (**Fig. 2**). Although both [4Fe-4S] and [2Fe-2S] clusters are found in several of the regulatory proteins discussed in this review, stable [3Fe-4S] clusters have thus far not been associated with regulatory activity, but such clusters are often involved in electron-transfer proteins (as are [4Fe-4S] and [2Fe-2S] clusters).

The acquisition of a [4Fe-4S] cluster by FNR results in conformational changes that reduce inter-subunit electrostatic repulsion, permitting homodimer formation, thereby enabling the C-terminal DNA-binding domain to recognize specific binding sites within target promoters.^10-13^ In *E. coli* K-12, FNR binds to 207 sites across the chromosome, most of which are associated with genes involved in anaerobic metabolism.^13^ In the presence of O_2_, the FNR [4Fe-4S]^2+^ cluster is converted into a [2Fe-2S]^2+^ form.^14,15^ This conversion results in FNR dimer dissociation, such that FNR neither binds DNA nor regulates gene expression.^16-19^ The [2Fe-2S]^2+^ cluster of FNR slowly degrades to form cluster-free (apo-) protein in the presence of O_2_ in vitro and in vivo.^20-22^ The apo-protein formed by cluster disassembly is capable of incorporating a new iron–sulfur cluster.^23-25^ However, the relative stability of the [2Fe-2S] form of FNR suggested that the [4Fe-4S] to [2Fe-2S] conversion could be reversed under some conditions. Initially, a fraction of [4Fe-4S]^2+^ cluster was observed after addition of dithionite to air-oxidized [2Fe-2S]^2+^ FNR in vitro.^15^ More recently, it has been recognized that bridging sulfide ions are retained within a persulfide coordinated [2Fe-2S] form of FNR and that this permits facile repair of the [4Fe-4S] cluster in the presence of ferrous ions and a reductant (**Fig. 2**).^26^ This suggests that the [2Fe-2S] form of FNR is not merely a passive intermediate in the conversion of the active [4Fe-4S] form to the inactive apo-form of FNR, but can act as a checkpoint allowing a return to active [4Fe-4S] form or further degradation to the apo-form depending on the prevailing O_2_ availability. Thus, O_2_ determines the transcriptional activity of FNR by promoting cycling of FNR between active [4Fe-4S], and inactive [2Fe-2S] and apo forms. This strategy requires that the concentration of FNR in the cell is held within a narrow range and this is the case in *E. coli* K-12.^20,24,27,28^

FNR is likely to be important for virulence of pathogens that encounter changes in O_2_ availability. In these cases, the absence of O_2_ sensed by FNR is thought to act as an environmental cue to reprogram metabolism, by activating genes required for anaerobic respiration (e.g., those encoding nitrate and nitrite reductases), fermentation (e.g., pyruvate formate-lyase, alcohol dehydrogenase) and trigger virulence gene expression during host colonization and infection. Accordingly, *Bordetella pertussis*, *Neisseria meningitidis*, *Pseudomonas aeruginosa*, and *Salmonella enterica* serovar Typhimurium (*S*. Typhimurium) FNR proteins were required for optimal growth and survival in vivo.^29-32^ Moreover, a proteomic analysis of *Shigella dysenteriae* type 1 supported the importance of a switch from aerobic respiration in vitro to anaerobic catabolism in vivo.^33^

As well as controlling the ability of many bacterial pathogens to adapt their metabolism to the hypoxic and anoxic niches within a host, FNR also contributes to regulating toxin production and effector protein secretion. Several strains of *E. coli*, *Salmonella*, and *Shigella* possess a cytolysin known as HlyE or ClyA.^34-39^ In *E. coli*, *hlyE* transcription is activated from a complex FNR-dependent class II promoter and HlyE activity is detected under anaerobic growth conditions.^40-45^ For these enteric bacteria, oxygen starvation could signal entry into a host and prompt expression of the HlyE cytolysin. In *Salmonella* Typhi, the causative agent of typhoid fever, *hlyE* mutants exhibited impaired invasion of human epithelial (HEp-2) cells and heterologous *hlyE* expression in *Salmonella* Typhimurium enhanced colonization of the spleen and liver in a mouse model of infection.^46^ The *Bacillus cereus*, non-hemolytic enterotoxin (Nhe) is a member of the HlyE family of pore-forming toxins and expression of *nhe* is under the control of the *B. cereus* FNR; however this control appears to be unresponsive to O_2_-availability.^47,48^ Although the *B. cereus* FNR has an O_2_-responsive [4Fe-4S] cluster, the cluster does not appear to be important for DNA-binding at the *nhe* promoter (there is evidence for monomeric apo-FNR binding) or for interaction with the redox-responsive regulator ResD (see below).^48-50^

**Figure 3.**
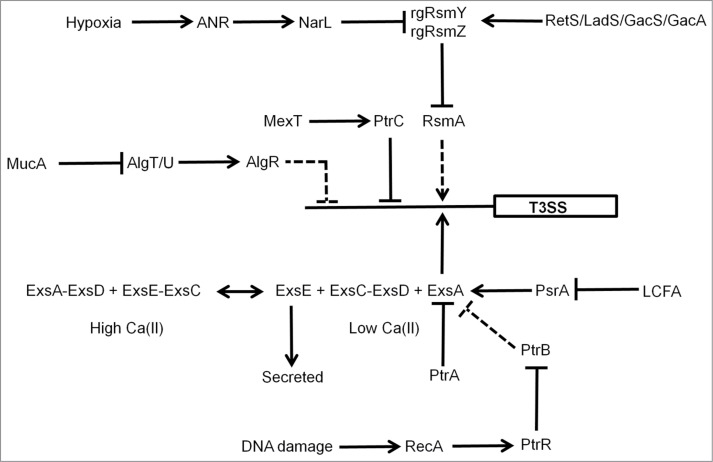
Regulation of the *P. aeruginosa* T3SS. Genetic regulation of the *P. aeruginosa* T3SS is complex. The O_2_ sensor, ANR, activates T3SS gene expression indirectly (dashed lines) via activation of *narL* gene expression under low O_2_ conditions and subsequent effects of the regulatory RNAs, rgRsmY and rgRsmZ on expression of the regulator RsmA. The activity of the rgRsmY and rgRsmZ regulatory RNAs is also controlled by the RetS/LadS/GacS/GacA cascade. The two-component system GacS–GacA is required for virulence in many hosts and phosphorylated GacA activates expression of rgRsmY and rgRsmZ. GacS is inhibited by formation of heterodimers with RetS and LadS activates the GacS–GacA system by an as yet unknown mechanism. In the presence of high Ca(II) concentrations, ExsE sequesters the anti-anti-activator ExsC, permitting the anti-activator ExsD to interact with the activator ExsA. Consequently, expression of the 43 genes required for the function of the T3SS is not activated. In the presence of low Ca(II) concentrations, ExsE is secreted. As a result ExsC sequesters ExsD, releasing ExsA to activate genes encoding the T3SS. ExsA-mediated activation is also antagonized by the anti-activator PtrA, by PtrB via the RecA response to DNA damage, and by PsrA (in response to long chain fatty acids, LCFA). The alginate regulators (MucA, AlgT/U, AlgR) act to repress T3SS expression indirectly. In addition, the efflux pump regulator MexT controls T3SS gene expression via the action of PtrC. T3SS genes are represented by a single rectangle. Arrows indicate activation, T-junctions indicate repression, solid lines indicate direct regulation, and dashed lines indirect regulation.

Oxygen-sensing by the *Shigella* FNR protein has been shown to play a role in coordinating the function of a Type III secretion system (T3SS) that is important for virulence. In the anaerobic lumen of the gastrointestinal tract, FNR primes the bacterium for invasion by activating expression of the T3SS needles, while repressing the expression of *spa32* and *spa33*, which regulate the function of the T3SS.^51^ Thus, the T3SS is built and ready to function as soon as *spa32* and *spa33* expression is triggered. As the *Shigella* approach the gut mucosa, they experience an increase in O_2_ availability, arising from the proximity to the capillary networks located in the villi. These micro-aerobic conditions result in FNR inactivation, by the mechanism discussed above, and the consequent de-repression of *spa32* and *spa33* allows invasion plasmid antigen secretion via the now functional T3SS precisely at its site of action.^51^ In *P. aeruginosa* the FNR protein (known as ANR) is a component of a regulatory network involving NarL and RsmAYZ that regulates the T3SS in response to host cells, low calcium and low O_2_ (**Fig. 3**).^52^ Moreover, the activity of ANR was stimulated under aerobic conditions by catabolism of choline and glycine-betaine that was generated from the breakdown of host membrane/lung surfactant phosphatidylcholine by hemolytic phospholipase C (PlcH), illustrating the complex relationships between O_2_ availability, FNR activity, metabolism, and virulence gene expression.^32^

## ArcBA, a Two-Component System That Senses O_2_ Indirectly

The ArcBA (**A**erobic **r**espiratory **c**ontrol) two-component system is an indirect sensor of O_2_ availability. ArcBA generally acts as a global regulator; it has been shown to control the expression of >175 genes in *E. coli* K-12, 392 genes in *S*. Typhimurium, 58 genes in the pig pathogen *Actinobacillus pleuropneumoniae*, but only 24 genes in *Haemophilis influenzae*. In all cases the core of the ArcBA regulon consists of genes associated with central metabolic and respiratory functions, such as those encoding enzymes of the Krebs cycle (e.g., *acnA*, *gltA*, *icd*, *fumA*, *mdh*, and *sdhCDAB-sucA-D* in *E. coli*) and the aerobic electron transport chain (e.g., *appCB*, *cydAB*, *cyoA-E*, and *nuoA-N* in *E. coli*) and thus, as with FNR, dysregulation of these key aspects of bacterial physiology is likely to lead to attenuation in the infective capacity of a pathogen. The absence of O_2_ results in reduction of components of the aerobic electron transport chain, including the quinone pool. The membrane-bound sensor, ArcB, responds to the redox state of the quinone pool via the oxidation state of two cysteine residues (in *E. coli* K-12, Cys-180, and Cys-241) located in a cytoplasmic PAS domain, such that in the absence of O_2_ the ArcB dimer undergoes autophosphorylation.^53-55^ Phosphoryl transfer from ArcB to the cytoplasmic regulator ArcA promotes ArcA oligomerization and DNA-binding to activate or repress the expression of target genes. In the presence of O_2_, the ArcB dimer acquires two inter-subunit disulfide bonds via interaction with the quinone pool, thereby inhibiting kinase activity and promoting ArcA dephosphorylation. As noted above, in most cases, ArcBA has been shown to be a global regulator of functions associated with central metabolism and fermentation, and thus dysregulation of these key physiological activities must contribute to the observed attenuation of *arcBA* mutants of *Klebsiella pneumoniae* in the colonization of gastrointestinal tract, and of *Shigella flexneri* plaque formation.^56,57^ ArcBA controls resistance to ROS and RNS in the highly virulent *S. enterica* serovar Enteritidis SE2472 strain, but the *arcBA* mutant was not attenuated in a mouse model of infection.^58^ However, conjugal transfer of the *Salmonella* virulence plasmid pSLT occurs at high frequency in the gastrointestinal tract and is dependent on ArcBA.^59^ In addition, ArcA has been shown to be a significant player in the regulation of: genes that are important for complement evasion in *Haemophilus influenzae*; the production of cholera toxin in *Vibrio cholerae* via regulation of *toxT*; and colonization of the porcine respiratory tract in *A. pleuropneumoniae*.^60-63^

## NO Resistance—Professional NO Sensors

Given the prominent role played by NO and RNS in the innate immune response to bacterial infection it is not surprising that pathogenic bacteria have evolved elaborate mechanisms to sense NO and respond to its presence through systems that detoxify NO and repair the damage caused by RNS. Although NO is an inhibitor of many heme enzymes that bind O_2_, some terminal oxidases are capable of contributing to NO detoxification by reduction of NO (**Fig. 1**). Moreover, some nitrite reductases, such as NrfA, can also reduce (detoxify) NO (see below; **Fig. 1**). Recently, a metabolomic screen to identify the effects of NO on the metabolism of *V. cholerae* revealed that NnrS is an NO-induced protein, which protects iron–sulfur proteins and the cellular iron-pool by lowering the production of dinitrosyl-iron complexes particularly under anaerobic conditions.^64^ However, the best characterized NO detoxification systems are the enzymes flavohemoglobin (Hmp) and flavorubredoxin (NorV).^4^ Hmp is primarily an NO dioxygenase, converting NO to NO_3_^−^, although it has limited anoxic NO denitrosylase activity producing NO^-^ (nitroxyl), which leads to the formation of N_2_O.^65^ Disruption of the *hmp* gene in *S*. Typhimurium severely impaired survival in macrophages. Uropathogenic *E. coli hmp* mutants were attenuated in a mouse urinary tract infection model, but a *P. aeruginosa hmp* mutant was not attenuated in a silk worm model.^66-69^ Hmp has an “on board” reductase system to supply electrons to the heme at the active site, but other bacterial globins that have been implicated in NO detoxification, such as those in *Campylobacter* (the Cgb globin) and *Mycobacterium* (the HbN globin) species, appear to lack a dedicated partner reductase, suggesting that turnover of NO by these proteins might be low, or that they are promiscuous, exploiting several cellular sources of reducing power.^70,71^ Nevertheless, the single domain hemoglobin (Cgb) of *Campylobacter jejuni* imparts NO resistance and expression of the *cgb* gene was induced upon exposure of the bacteria to RNS.^72,73^ A prominent anaerobic/hypoxic NO detoxification system in *E. coli* K-12 is NorV (along with its dedicated reductase NorW), which catalyzes the reduction of NO to NO^−^ (and ultimately N_2_O).^74^ Inactivation of *norV* (by truncation of the gene, which occurs in some natural isolates) of enterohemorrhagic *E. coli* O157 revealed an important role for the intact *norV* gene in macrophage survival and was thus considered to be a direct virulence determinant.^75^ Recently, a new class of NO reductase (represented by the Hp0013 protein) has been recognized in *Helicobacter pylori*.^76^ The *H. pylori hp0013* mutant is more sensitive to NO and is defective in colonization of the stomachs of mice.^76^ Bacteria that are capable of denitrification, (i.e., the stepwise reduction of nitrate to nitrogen gas via nitrite, NO and nitrous oxide; **Fig. 1**), possess NO reductase enzymes that catalyze the formation of N_2_O from NO.^76,77^ Abolishing this activity impairs the virulence of *P. aeruginosa*.^69^ A further route to NO detoxification under anoxic conditions is via the action of cytochrome *c* nitrite reductase (NrfA), which although it has a high K_m_ for NO has a high turnover rate and alongside NorV accounts for most of the anaerobic NO reductase activity in *S*. Typhimurium.^78,79^

As well as detoxification of NO, bacteria also respond by inducing mechanisms to repair damaged cell components. Although little information is available on these processes, it appears that the YtfE protein contributes to the repair of nitrosylated iron–sulfur clusters and Ogt has a role in DNA repair in *E. coli*. It has been suggested that the NO-regulated *hcp-hcr*, *yeaR*, and *yoaG* gene products have as yet uncharacterized roles in repairing NO damage.^80^

The bacterial responses to NO discussed above are mostly regulated at the level of transcription by NO-responsive transcription factors, some of which are considered below.

### NsrR

NsrR is a member of the Rrf2 family of transcription factors and is found in most β and γ proteobacteria, notable exceptions in the current context being the *Pasteurellaceae*, Pseudomonadales, and *V. cholerae*.^81,82^ The *E. coli* NsrR protein controls the expression of >60 genes, including *hmp*.^83^ The NsrR regulon of *S*. Typhimurium overlapped that of *E. coli* and several of the gene products were shown to be important for growth during nitrosative stress (i.e., the stress/damage imposed on a biological system by exposure to NO and its congeners derived from the initial reaction of NO with superoxide).^84^ NsrR from *Neisseria gonorrhoeae*, the causative agent of the sexually transmitted disease gonorrhea, is a [2Fe-2S] protein with three conserved C-terminal Cys residues that act as cluster ligands; the identity of the fourth coordinating residue is unknown although a conserved His residue has been suggested to fulfill this role.^85^ DNA-binding by *N. gonorrhoeae* NsrR was abolished by exposure to NO, presumably due to nitrosylation of the iron–sulfur cluster.^85^ The *Streptomyces coelicolor* NsrR protein possesses an O_2_-stable [2Fe-2S] cluster that reacts with NO to yield a dinitrosyl-iron complex and this form of the protein could not bind to target DNA.^86^ Thus, the *S*. *coelicolor* and *N. gonorrhoeae* NsrR proteins have similar properties. However, although the DNA-binding activity of the NsrR protein of the non-pathogen *B. subtilis* was sensitive to NO, this protein apparently possesses a [4Fe-4S] cluster.^87,88^ Hence, there is some uncertainty about the nature of the NsrR iron–sulfur cluster and therefore the mechanism by which NO modulates the transcriptional activity of NsrR.

### NorR

**Figure 4.**
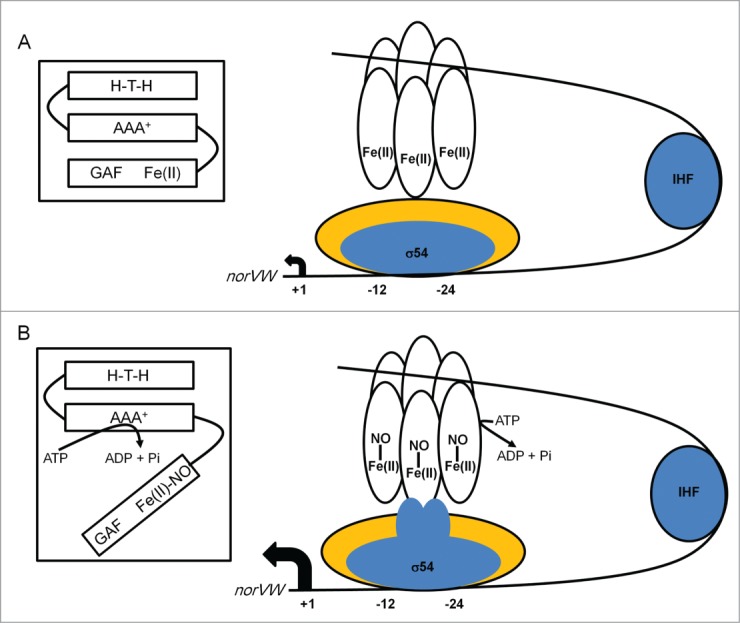
Scheme summarizing the action of NorR at the *E. coli norVW* promoter. (**A**) In the absence of NO hexameric NorR (unfilled ovals) is able to bind to enhancer elements located upstream of the *norVW* core σ^54^-dependent promoter elements (-12 and -24) via its helix-turn-helix (H-T-H) DNA-binding domain. Integration host factor (IHF) bends the DNA such that NorR and the σ^54^-RNA polymerase holoenzyme can potentially interact. However, these interactions are unproductive because the ATPase activity of the NorR AAA^+^ domain is inhibited by interaction with the GAF domain, which contains the sensory mononuclear iron center (Fe[II]) (see inset). Consequently, *norVW* transcription is switched off (small filled arrow, +1). (**B**) When NO binds at the mononuclear iron centers of NorR (Fe[II]-NO) the AAA^+^ domain is released from the sensory GAF domain (see inset) and acquires ATPase activity allowing productive interactions with σ^54^-RNA polymerase. The ensuing conformational changes promote the formation of the open complex and enhance *norVW* transcription (large filled arrow, +1). For clarity, not all the regulatory elements operating at this promoter are shown. The diagram is not drawn to scale.

NorR is a σ^54^-dependent transcriptional regulator with an N-terminal GAF domain, an AAA^+^ ATPase domain and a C-terminal helix-turn-helix DNA-binding domain (**Fig. 4**). GAF is a common small-molecule binding domain that is distantly related to another ligand binding domain PAS (see ArcA above). In NorR the GAF domain houses a non-heme iron center that reversibly binds NO.^89,90^ In the absence of NO the GAF domain sequesters the AAA^+^ domain, inhibiting ATPase activity and productive interaction with σ^54^-RNA polymerase. The non-heme iron is thought to be hexa-coordinate and ligated by 5 amino acids (Arg-75, Asp-96, Asp-99, Cys-113, and Asp-131). Reaction with NO results in the formation of a mononitrosyl iron complex and the concomitant liberation of the AAA^+^ domain allowing the AAA^+^ domain to make productive interactions with the σ^54^ subunit of RNA polymerase and activate transcription of *norVW*, encoding the NorVW NO reductase.^91^ The *norVW* promoter has three tandem enhancer sites that are essential for NorR ATPase activity.^92^ In *E. coli* the *norVW* operon is the only known target for NorR, but in *P. aeruginosa* and *V. cholerae*, which lack NsrR, NorR activates *hmp* expression, and in a mouse prolonged colonization model a *V. cholerae norR* mutant was attenuated.^93^

### NssR

The major cause of gastroenteritis in developed countries is chicken contaminated with *Campylobacter* species. After ingestion, the bacteria are exposed to NO and other RNS generated by the host immune system and from acidification of the nitrite in saliva and the nitrite generated by the reduction of dietary nitrate. In *C. jejuni* the cyclic-AMP receptor protein (CRP) family regulator NssR controls the expression of a small regulon, including those encoding two globins Cgb and Ctb (see above).^72^ NssR acts as a positive regulator of both *cgb* and *ctb* under nitrosative stress conditions, but high-affinity DNA-binding by NssR was unaffected by NO, suggesting that NssR-mediated activation of *cgb* and *ctb* occurs downstream of DNA-binding.^94^ The mechanism by which NssR senses the presence of NO is unknown, although it has been noted that the protein has a single cysteine that could be a target for nitrosylation or one or more tyrosine residues might be nitrated by peroxynitrite.^94^

## NO Resistance—Secondary NO Sensors

### FNR

The *E. coli* FNR [4Fe-4S] cluster reacts not only with O_2_ (see above) but also with NO (**Fig. 2**).^95-97^ Reaction with NO is extremely rapid, multiphasic and results in the formation of a protein-bound nitrosylated iron–sulfur cluster that resembles a pair of Roussin's red esters.^97^ Reaction with NO inhibits FNR DNA-binding activity in vitro and FNR-dependent transcription in vivo.^95,97^ Thus, in addition to its well-established role as an O_2_-responsive regulator of anaerobic functions, the inactivation of FNR by NO was suggested to be a final safeguard against NO toxicity by switching off transcription of genes involved in nitrate and nitrite respiration, thereby minimizing endogenous NO production when the dedicated NO-responsive regulators and detoxification systems are overwhelmed.^97^

### SoxR

The SoxRS system of enteric bacteria consists of two DNA-binding proteins, which act sequentially to regulate the transcription of >100 genes in response to redox stress caused by exposure to superoxide and/or bacteria- and plant-derived redox-cycling molecules, such as pyocyanin and plumbagin.^98–101^ This regulon includes genes encoding proteins involved in detoxification of ROS (superoxide dismutase), repair of ROS-mediated damage (endonuclease IV), and replacement of ROS-sensitive components by resistant ones (fumarase C). Thus, the SoxRS regulon contributes to resisting the toxic effects of the macrophage oxidative burst. Although SoxR is widely distributed, SoxS is absent in non-enteric bacteria, and in these cases SoxR is responsible for regulating all members of the regulon.^102^ The SoxRS and SoxR systems have been associated with fluoroquinolone resistance in *Salmonella* serovars, the ability of *P. aeruginosa* to survive in macrophages, cause systemic infections following burn wounds and cause pulmonary infections, and virulence of *Vibrio vulnificus* and *Xanthomonas campestris*.^103–107^ SoxR is a homodimeric, MerR family protein.^107^ Each monomer has a cluster of four cysteine residues (Cys-X_2_-Cys-X-Cys-X_5_-Cys) that binds a solvent-exposed [2Fe-2S]^1^^+^ center in an asymmetric electrostatic environment.^108^ All forms of SoxR bind to target DNA, but it is the one-electron oxidation of the [2Fe-2S]^1^^+^ form of SoxR that generates the transcriptionally active [2Fe-2S]^2^^+^ form. Furthermore, DNA contributes to setting the sensitivity of the SoxR switch, shifting the reduction potential from −285 mV for SoxR in solution to +200 mV for SoxR bound to its cognate DNA.^109^ Transcriptional activation occurs by remodeling the −35 and −10 promoter elements.^108^ Upon activation, SoxR activates transcription of *soxS*, and the SoxS protein switches on expression of the SoxRS regulon. The system is switched off by a SoxR reductase, encoded by *rseC* and *rsxABCDGE*, and by proteolytic degradation of SoxS.^110,111^

As well as responding to redox-cycling molecules, the *E. coli* SoxR [2Fe-2S] cluster reacts with NO to form a protein-bound dinitrosyl-iron complex that activates expression of *soxS* and hence the SoxRS regulon.^112–115^ The activation of the SoxRS system by NO conferred resistance to activated macrophages and was thus considered important in virulence.^112^ Thus, although the primary role of SoxR is to sense and respond to oxidative stress, it may play a significant secondary role in the response to nitrosative stress.

### OxyR

**Figure 5.**
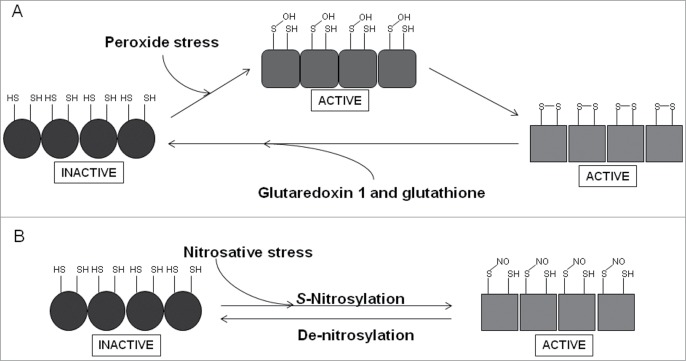
Scheme summarizing the redox-reactivity of OxyR. (**A**) The sensory C-terminal domain of each monomer in the OxyR homotetramer contains a redox-reactive cysteine residue (Cys-199), which forms a sulfenic acid (S-OH) in the presence of peroxide stress. This form of OxyR is proposed to be able to regulate gene expression, although more likely acts as an intermediate in forming the true active form, which is able to bind DNA and serve as a transcriptional regulator and contains an intra-molecular disulphide bond between Cys-199 and Cys-208. OxyR returns to its inactive form (Cys-199, SH; Cys-208, SH) by the action of glutaredoxin 1 and glutathione. (**B**) A secondary role of OxyR is as a nitrosative stress responder. *S*-nitrosylation of Cys-199, forming S-NO, leads to activation of OxyR, de-nitrosylation, forming SH, returns OxyR to its inactive form.

OxyR is a member of the LysR family of transcription factors and is responsible for coordinating the response to peroxide stress in many bacteria. In *E. coli*, OxyR controls a regulon that includes the sRNA *oxyS* and genes encoding proteins for the detoxification of peroxides (catalase, alkylhydroperoxidase), for the repair of damaged cell components (methionine sulfoxide reductase) and protection of DNA (Dps).^116^ OxyR exists as a homotetramer, with each subunit possessing two domains; an N-terminal DNA-binding domain and a C-terminal sensory domain (**Fig. 5**).^117^ The latter contains the redox-reactive cysteine residue (Cys-199), which in the presence of peroxide stress forms a sulfenic acid (Cys-199, S-OH) that is apparently sufficient to activate OxyR, but there is good structural and biochemical evidence that the active form of OxyR has an intra-subunit disulfide bond linking Cys-199 and Cys-208; thus the sulfenic acid form is likely to be an intermediate in the production of the disulfide form.^117,118^ Upon oxidation, OxyR recruits RNA polymerase to target promoters to activate transcription, or represses gene expression by promoter occlusion. OxyR is switched off when redox balance is restored by the action of glutaredoxin 1 (an OxyR target) and glutathione. Not surprisingly, OxyR is considered to be important in co-ordinating the response to ROS generated during the oxidative burst of macrophages and has been shown to be critical for full virulence of many bacterial pathogens. For example, OxyR has been shown to contribute to the virulence of *Bacteroides fragilis*, *E. coli*, *Francisella novicida*, *K. pneumoniae*, *P. aeruginosa*, *Ralstonia solanacearum*, *X. campestris*, and *Y. pestis*, but not *Mycobacterium marinum* or intestinal colonization by *S. enterica*.^119–129^ In addition to its primary role in response to peroxide stress, OxyR is activated by nitrosative stress as a result of *S*-nitrosylation (Cys-199, S-NO); de-nitrosylation (Cys-199, SH) inactivates OxyR.^118,130^ *S*-Nitrosylation of OxyR induced expression of a set of genes, distinct from those activated in response to oxidative stress, which limited *S*-nitrosylation of proteins and thereby contributed to protection from nitrosative stress.^131^

## Oxygen and NO Sensing in *Staphylococcus aureus*

*Staphylococcus aureus* is carried on the skin and mucosa (anterior nares) by up to 20% of the population at any one time without any harmful effects. However, it is an opportunistic pathogen that is capable of causing a range of diseases including bacteremia, chronic lung infections, endocarditis, food poisoning, meningitis, osteomyelitis, skin infections, and wound infections, and is one of the most common causes of hospital acquired infection.^132^ The bacterium is a facultative anaerobe and the ability to adapt to anoxic conditions and mount a defense against host-generated NO is vital in the pathogenesis of many of these diseases. Despite this, the mechanisms that enable *S. aureus* to perceive and respond to changes in the availabilities O_2_ and NO are poorly understood. In this section the roles of three staphylococcal two-component regulators in these processes are reviewed.

### SrrAB

**Figure 6.**
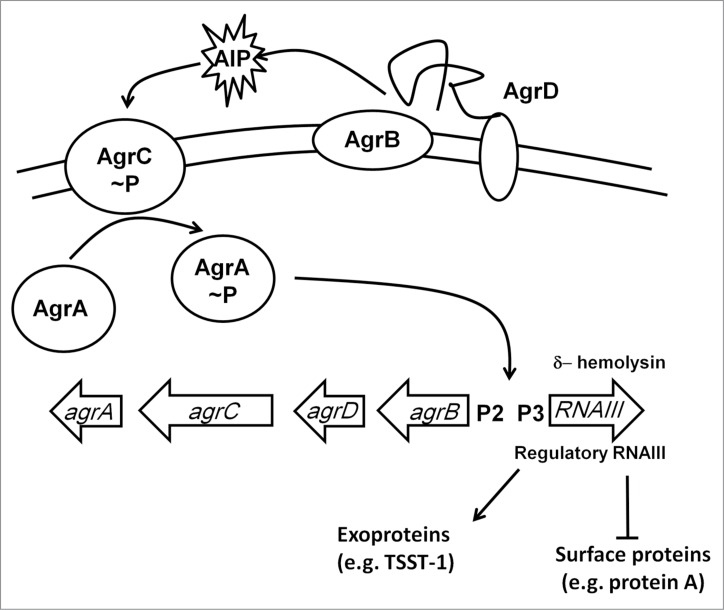
The Agr regulatory system. The *agr* locus consists of divergently transcribed *agrBDCA* and RNAIII genes. The former is driven from promoter 2 (P2) and encodes proteins that constitute the Agr quorum sensing system. The latter is driven from P3 and encodes the 26 amino acid δ-hemolysin and the regulatory RNA, RNAIII. AgrC and AgrA are a two-component system that responds to accumulation of an autoinducer peptide (AIP, a tailed thiolactone ring) that is generated by processing of AgrD by the membrane-bound AgrB protein and SpsB. The accumulation of AIP in the extracellular milieu is sensed by AgrC resulting in phosphorylation and activation of AgrA. RNAIII downregulates expression of cell surface proteins and upregulates exoprotein (toxin) production.

The two-component system ResDE is required for anaerobic respiration in many gram-positive bacteria; in *Staphylococci* the ResDE othologs are known as SrrAB.^133^ The ResE (SrrB) protein is a membrane-anchored sensor that autophosphorylates in the absence of O_2_ and then transfers the phosphate to the cytoplasmic response regulator ResD (SrrA). The precise signal sensed by these systems is unknown; it is unlikely to be O_2_ per se but more likely a physiological consequence of O_2_-starvation, such as changes in the redox state of the electron transport chain (see ArcBA above). Under anaerobic conditions, SrrAB downregulates *agr*-RNAIII, a regulatory RNA that enhances the production of secreted virulence factors such as serine protease and α-hemolysin, and inhibits the synthesis of cell-surface proteins such as protein A (**Fig. 6**).^134^ SrrAB also downregulates synthesis of the toxic shock syndrome toxin 1 (TSST1) and enhances transcription of the *ica* operon resulting in increased production of extracellular polysaccharide.^135,136^ A strain of *S. aureus* that overexpressed *srrAB* was attenuated in a rabbit model of endocarditis by ∼100-fold, presumably due to the repression of major virulence factors such as *agr*-RNAIII, TSST1, and protein A, and hence O_2_-sensing (probably indirectly) by SrrAB modifies the virulence of *S. aureus*.^136^

### NreABC

*Staphylococcus aureus* can utilize O_2_, nitrate, or nitrite as a terminal electron acceptor. However, unlike the enteric bacteria in which regulation of genes required for anaerobic respiration is coordinated by the global O_2_-sensing transcription factor FNR, in the staphylococci the regulation of nitrate-nitrite respiration is assumed by the proteins encoded by the *nreABC* operon.^137^ The NreBC proteins constitute a two-component system; however, how NreA impacts on the activity of NreBC is unknown, but NreA has a GAF domain and is thought to be involved in sensing nitrate. NreB is a cytoplasmic histidine kinase with four Cys residues located within an N-terminal PAS domain that binds a [4Fe-4S] cluster. Like FNR, the NreB iron–sulfur cluster is disassembled in the presence of O_2_, such that in the absence of O_2_ the kinase activity of NreB is activated.^138,139^ Thus, in the absence of O_2_ NreB phosphorylates the response regulator, NreC, which is then competent for site-specific DNA-binding to activate expression of at least 40 genes including the anaerobic respiratory *nar* and *nir* operons, genes involved in nitrogen metabolism, fermentation, and biofilm formation.^138–140^

An *S. aureus narJ* mutant emerged from a large-scale (6300 insertion mutants) screening experiment for strains attenuated in a mouse model of systemic infection, but this strain was similarly attenuated in vitro and hence probably has a general growth defect.^141^ Thus, the evidence indicates that NreABC does not play a major role in the control of virulence gene expression in response to hypoxia but is important as a fitness factor in anoxic environments where nitrate is available, which could be relevant to infection. Accordingly, O_2_ availability has been suggested to control the spatial and temporal expression of Cid, an autolysin that contributes to the provision of extracellular DNA in the biofilm matrix by controlling bacterial programmed cell death, because the *cidABC* operon was induced under the hypoxic conditions that exist in the interior of the tower structures in biofilms.^142^ This link between NreABC regulation and biofilm growth and maturation is potentially important because *S. aureus* is one of the most frequent causes of biofilm-associated infection on indwelling medical implants.

### AirSR

A third *S. aureus* two-component system, AirRS (formerly YhcRS), acts as a global regulator under anoxic conditions and controls, directly or indirectly, the expression (both up- and downregulation) of >350 genes, including the Agr regulatory system (**Fig. 6**) and virulence factors such as capsular polysaccharide synthesis (*cap5A*), protein A (*spa*), leukotoxin (*lukD*), and γ-hemolysin (*hlgC*) in the Newman strain, and *nreABC* (see above) as well as several metabolic genes, which could be important for virulence in the WCUH29 isolate, in which AirSR appears to be essential.^143,144^ The N-terminal region of the histidine kinase AirS has a cysteine cluster (Cys-X_7_-Cys-X-Cys-X_17_-Cys) that acts as the locus for a [2Fe-2S] cluster. The AirS iron–sulfur cluster reacted relatively slowly with O_2_ as demonstrated by the fact that the protein could be isolated with a [2Fe-2S] cluster under aerobic conditions; however, the cluster reacted more rapidly with hydrogen peroxide, resulting in cluster degradation. The cluster also reacted with the nitrosating agent *S*-nitrosoglutathione to yield a protein bound dinitrosyl-iron-dithiol complex.^143^ Oxidative degradation of the [2Fe-2S] cluster to form apo-AirS or formation of the nitrosylated cluster inhibited the kinase activity of AirS and consequently interrupted transfer of phosphate to the response regulator AirR, switching off expression of the AirRS regulon.^143^ The phenotypic consequences of disruption of the AirRS system are increased resistance to H_2_O_2_, vancomycin, norfloxacin, and ciprofloxacin under anaerobic conditions.^143^

### Nitric oxide responses

The relationship between *S. aureus* and NO is more complex than that described above for enteric pathogens because *S. aureus* is one of a few gram-positive bacteria that possess a nitric oxide synthase.^145–147^ In several of these bacteria the capacity to synthesize NO has been shown to contribute to bacterial virulence, increase resistance to oxidative stress and provide protection against antibiotics, and, in the case of *Streptomyces sturgidiscabies*, nitration is required to activate a phytotoxin.^147–149^ The *S. aureus* nitric oxide synthase also protects against killing by neutrophils, as well as being involved in the development of skin abscesses in a mouse model.^150–152^ *Staphylococcus aureus* is tolerant to nitrosative stress by transcriptional reprogramming that involves at least 84 identified genes, many of which have roles in iron-homeostasis and hypoxic metabolism; the latter falling under the influence of the indirect O_2_-responsive SrrAB two-component system (see above). Consequently, an *srrAB* mutant exhibited enhanced sensitivity to NO and this was partially attributed to dysregulation of the NO detoxification enzyme Hmp (see above), but the divergently transcribed *ldh1* gene, encoding lactate dehydrogenase was subsequently shown to be essential for virulence and maintaining redox balance during nitrosative stress.^153,154^ The overlap between the response to anoxia and exposure to NO could be accounted for by NO-mediated inhibition of aerobic respiration triggering the activation of the SrrAB two-component system and consequently the role of SrrAB could be extended beyond the control of hypoxic metabolic and major virulence factor genes to include NO resistance; a combination that readily explains the attenuation of the *srrAB* mutant.

At the time of writing, the only *S. aureus* gene regulator that reacts directly with NO is the AirSR two-component system (see above). Nitrosylation of the [2Fe-2S] cluster of AirS by NO inhibits its histidine kinase activity and hence switches off the regulatory activity of AirR.^143^ Further work is needed to establish whether NO is a physiological signal for the AirSR system, but at this stage it seems to be a good candidate.

## The Response of *Mycobacterium tuberculosis* to O_2_ and NO

*Mycobacterium tuberculosis* is the causative agent of tuberculosis (TB) in humans and infects up to one-third (∼2 billion) of the world's population, of which 5–10% are at risk of developing active TB.^155^ Fortunately, most infected individuals are essentially asymptomatic, carrying the bacteria in lung lesions, known as tubercules. Exposure to hypoxia and NO in the tubercule, reprograms *M. tuberculosis* gene expression to facilitate entry into a non-replicative, drug-resistant, persistent state.^155–161^ In this state, known as latency, the bacteria survive for decades in the infected lung, before potentially emerging as an active TB infection when an individual becomes immune-compromised.^156–159^ Among the environmental cues that trigger transition to dormancy within the host are hypoxia and exposure to NO.^161,162^ Therefore, sensing and responding to these signals is a central feature of *M. tuberculosis* virulence and TB pathogenesis. The sensory mechanisms and roles of some of the key transcription regulators involved in this process are discussed below.

### DosR/S/T

As noted above *M. tuberculosis* is exposed to NO and hypoxia during the course of an infection. Adaption in response to these signals is mediated by the three-component dormancy survival regulator (DosR/S/T). The two sensor kinases, DosS and DosT possess tandem GAF domains, the first of which (GAF-A) contains a penta-coordinate ferrous-heme that interacts with NO, O_2_, and CO, followed by histidine kinase and ATPase domains.^163–165^ Although it has been proposed that DosS is a redox sensor and DosT a hypoxia sensor, it is mostly likely that both are gas sensors.^166^ The deoxy-ferrous forms of DosS and DosT autophosphorylate in the absence of O_2_ or when NO (or CO) binds at the sensory heme; binding of O_2_ inhibits autophosphorylation as a result of conformational changes initiated by hydrogen-bonding network involving O_2_-bound to heme and a conserved Tyr residue.^166^ The inactive oxy-heme-form of DosS is readily converted to the active ferrous-NO-form in the presence of low concentrations of NO, activating the DosR regulon.^167^ Phosphorylated DosS/T transfers phosphate to DosR, activating DNA-binding and initiating the dormancy gene expression program, which includes *dosS*. Despite their similarity, DosS and DosT appear to play distinct roles, the former acting in final phase and the latter in the initial phase of the transition to dormancy, and they exhibit some differences in ligand binding; notably that DosT traps O_2_ better than DosS.^166^^,^^168–170^

In response to hypoxia, non-toxic concentrations of NO and adaptation to an in vitro dormant state DosR controls the expression of a common set of ∼50 identified genes.^162^^,^^171–174^ Among these genes are those involved in controlling the shift from aerobic to anaerobic metabolism, allowing the bacilli to survive during hypoxia-induced dormancy, and be positioned to return to replication (and thus active infection) upon re-oxygenation.^174–177^ This permits the bacteria to enter dormancy, aiding survival, when conditions are unfavorable for active infection. The individual contributions of many genes induced by the Dos R/S/T regulon in helping *M. tuberculosis* survival during dormancy (persistence factors), remain unclear. Nevertheless, the essential role played by the Dos system in the ability of *M. tuberculosis* to establish and emerge from dormancy is a major contributor to TB pathogenesis, allowing the establishment of an enormous reservoir of infection.

### WhiB-like proteins

*Mycobacterium tuberculosis* possesses seven WhiB-like (Wbl) proteins. Wbl proteins are found exclusively in the actinomycetes and play important roles in developmental processes.^178^ All Wbl proteins have four highly conserved cysteine residues, with the central two forming a CXXC motif in the majority of the members, and a weakly predicted helix-turn-helix in the C-terminal region.^179^ These two key features suggest that Wbl proteins bind a metal co-factor, which senses and responds to environmental signals to modulate DNA-binding via the C-terminal region. Accordingly, several Wbl proteins from *S. coelicolor* and *M. tuberculosis* have been identified as iron–sulfur proteins that are redox, O_2_, and/or NO-sensitive.^180–188^ Furthermore, conditional DNA-binding activity has been demonstrated in several cases.^184–186^ The best characterized *M. tuberculosis* Wbl proteins are WhiB1 and WhiB3.

The *M. tuberculosis whiB1* gene is essential and the conserved cysteine residues of the encoded protein coordinate a [4Fe-4S]^2^^+^ cluster, which unlike that of FNR (see above) is stable in the presence of O_2_.^186,188^ However, like FNR, the WhiB1 iron–sulfur cluster reacts rapidly with 8 molecules of NO, forming an octa-nitrosylated cluster.^188^ Reaction of holo-WhiB1 with NO converts WhiB1 from a non-DNA-binding form to a form capable of binding both the *whiB1* and *groEL2* (encoding an essential chaperonin) promoters, and repressing transcription of both genes in vitro.^186,189^ Repression of *groEL2* expression by WhiB1 might contribute to inhibiting *M. tuberculosis* growth during the NO-induced transition to the persistent non-replicating state that is characteristic of latent tuberculosis infections. DNA-binding activity was also observed with both the oxidized (disulfide form) and reduced (dithiol form) apo-WhiB1. Thus the presence/absence and state of the iron–sulfur cluster, and the oxidation state of cysteine residues in apo-WhiB1, govern the ability of WhiB1 to bind DNA via its C-terminal region.^186,187^ The full extent of the WhiB1 regulon is currently unknown, but its role as an essential, aerobic NO-sensing transcription factor implies that WhiB1 and the genes that it controls are likely to contribute to transcriptional reprogramming in the host environment.^186^

WhiB3 from *M. tuberculosis* is the best studied of the Wbl proteins and like WhiB1, possesses a [4Fe-4S] cluster and is a DNA-binding protein that controls several aspects of virulence, including the biosynthesis of complex surface-associated virulence lipids.^185^^,^^190–192^ The expression of *whiB3* is enhanced in macrophages and the mouse lung, indicating that *M. tuberculosis* regulates *whiB3* expression in response to environmental signals associated with infection; this is supported by the findings that hypoxia and NO induced *whiB3* expression.^193–196^ Its role in virulence is clear—mice infected with a *whiB3* null mutant showed increased survival.^186^ Moreover, WhiB3 directly controls expression of genes involved in the biosynthesis of the secreted, immuno-modulatory lipids, poly- and di-acyltrehaloses, sulfolipids, and phthiocerol dimycocerosates that are associated with persistence and latency, as well as the storage lipid triacylglycerol (TAG). The iron–sulfur form of WhiB3 (holo-WhiB3), in both reduced and oxidized states, binds DNA very weakly, but the oxidized (disulfide form) of apo-WhiB3 exhibits strong DNA-binding activity.^192^ The WhiB3 iron–sulfur cluster reacts with both O_2_ and NO, probably via mechanisms resembling that for FNR and WhiB1 (see above).^185^ It has been suggested that these responses indicate that under the oxidizing aerobic conditions associated with active TB infections apo-WhiB3 is transcriptionally active, whereas under the reducing hypoxic conditions associated with latency WhiB3 possesses an iron–sulfur cluster and is transcriptionally inactive. Hence WhiB3 senses the redox state of the bacterium via the presence/absence of the iron–sulfur cluster and the propensity of the cysteine residues that ligate the iron–sulfur cluster to form intramolecular disulfide bonds under oxidizing conditions.^185,191^ The physiological significance of the NO-reactivity of the WhiB3 iron–sulfur cluster has yet to be established.

## Conclusions

**Figure 7.**
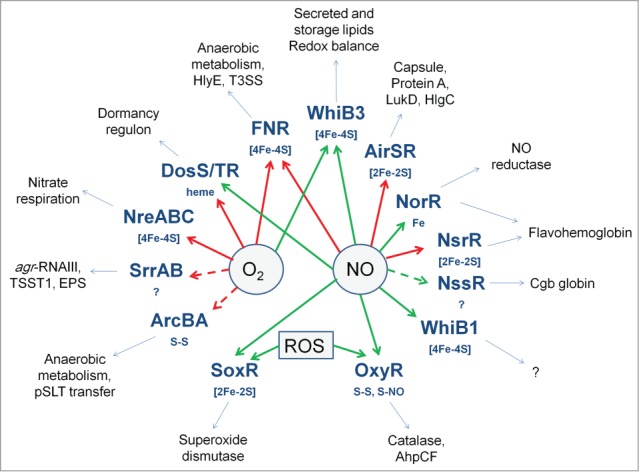
Oxygen- and NO-responsive bacterial transcription factors. The transcription factors with their sensory co-factors (if these are known) are shown in bold type-face. Direct sensing of O_2_ or NO is indicated by solid arrows; indirect or unknown sensing mechanisms by broken arrows; red arrows indicate that the signal molecule inhibits DNA-binding; green arrows indicate that the signal molecule promotes DNA-binding. Examples of virulence-related processes, toxins, cell-structural components, and proteins that are regulated by the transcription factors are shown in the outer ring.

The ability to sense and respond to changes in O_2_ availability and exposure to the toxic gas NO is crucial for many bacterial pathogens. Both these gases can act as environmental cues to reprogram gene expression and thereby promote the ability to grow and replicate within a host (e.g., switching from aerobic to anaerobic metabolism and synthesizing systems for NO detoxification), to activate expression of virulence factors to attack a host (e.g., T3SS, HlyE, LukD, TSST1) and, in the case of *M. tuberculosis* and possibly other pathogens, facilitate entry into a persistent, non-replicating state (**Fig. 7**). Several transcription factors involved in O_2_ sensing are global regulators (e.g., ArcBA, FNR, SrrAB) controlling key aspects of central metabolism, as well as genes encoding virulence factors (**Fig. 7**). This suggests that these core regulators have been evolutionarily co-opted to coordinate virulence gene expression with the metabolic adaptations triggered by host-associated hypoxia. On the other hand NO-responsive transcription factors appear to play more specialized roles associated with NO detoxification and redox homeostasis (**Fig. 7**). However, regulators such as DosS/T/R, WhiB3, and FNR can act as sensors of both O_2_ and NO, raising questions of how their respective sensory co-factors react with, and discriminate between, these similar gases to trigger different patterns of gene expression. Research targeted toward obtaining a deeper understanding of the interplay between differential signal perception and the transcriptional outputs resulting from the action of multiple regulators acting at the promoters of virulence genes should provide new paradigms in host-pathogen interaction by defining the transcriptional colloquy that is crucial in determining the outcome of an infection.

## References

[1] Imlay JA. Pathways of oxidative damage. Annu Rev Microbiol 2003; 57:395-418; PMID:14527285; http://dx.doi.org/10.1146/annurev.micro.57.030502.090938 14527285

[2] Imlay JA. Cellular defenses against superoxide and hydrogen peroxide. Annu Rev Biochem 2008; 77:755-76; PMID:18173371; http://dx.doi.org/10.1146/annurev.biochem.77.061606.161055 18173371 PMC3057177

[3] Robinson MA, Baumgardner JE, Otto CM. Oxygen-dependent regulation of nitric oxide production by inducible nitric oxide synthase. Free Radic Biol Med 2011; 51:1952-65; PMID:21958548; http://dx.doi.org/10.1016/j.freeradbiomed.2011.08.034 21958548

[4] Bowman LA, McLean S, Poole RK, Fukuto JM. The diversity of microbial responses to nitric oxide and agents of nitrosative stress close cousins but not identical twins. Adv Microb Physiol 2011; 59:135-219; PMID:22114842; http://dx.doi.org/10.1016/B978-0-12-387661-4.00006-9 22114842

[5] Crack JC, Green J, Thomson AJ, Le Brun NE. Iron-sulfur cluster sensor-regulators. Curr Opin Chem Biol 2012; 16:35-44; PMID:22387135; http://dx.doi.org/10.1016/j.cbpa.2012.02.009 22387135

[6] Crack JC, Green J, Hutchings MI, Thomson AJ, Le Brun NE. Bacterial iron-sulfur regulatory proteins as biological sensor-switches. Antioxid Redox Signal 2012; 17:1215-31; PMID:22239203; http://dx.doi.org/10.1089/ars.2012.4511 22239203 PMC3430481

[7] Green J, Crack JC, Thomson AJ, LeBrun NE. Bacterial sensors of oxygen. Curr Opin Microbiol 2009; 12:145-51; PMID:19246238; http://dx.doi.org/10.1016/j.mib.2009.01.008 19246238

[8] Sharrocks AD, Green J, Guest JR. *In vivo* and *in vitro* mutants of FNR the anaerobic transcriptional regulator of *E. coli.* FEBS Lett 1990; 270:119-22; PMID:2226775; http://dx.doi.org/10.1016/0014-5793(90)81248-M 2226775

[9] Green J, Sharrocks AD, Green B, Geisow M, Guest JR. Properties of FNR proteins substituted at each of the five cysteine residues. Mol Microbiol 1993; 8:61-8; PMID:8497198; http://dx.doi.org/10.1111/j.1365-2958.1993.tb01203.x 8497198

[10] Moore LJ, Mettert EL, Kiley PJ. Regulation of FNR dimerization by subunit charge repulsion. J Biol Chem 2006; 281:33268-75; PMID:16959764; http://dx.doi.org/10.1074/jbc.M608331200 16959764

[11] Green J, Irvine AS, Meng W, Guest JR. FNR-DNA interactions at natural and semi-synthetic promoters. Mol Microbiol 1996; 19:125-37; PMID:8821942; http://dx.doi.org/10.1046/j.1365-2958.1996.353884.x 8821942

[12] Scott C, Partridge JD, Stephenson JR, Green J. DNA target sequence and FNR-dependent gene expression. FEBS Lett 2003; 541:97-101; PMID:12706827; http://dx.doi.org/10.1016/S0014-5793(03)00312-0 12706827

[13] Myers KS, Yan H, Ong IM, Chung D, Liang K, Tran F, Keleş S, Landick R, Kiley PJ. Genome-scale analysis of *escherichia coli* FNR reveals complex features of transcription factor binding. PLoS Genet 2013; 9:e1003565; PMID:23818864; http://dx.doi.org/10.1371/journal.pgen.1003565 23818864 PMC3688515

[14] Jordan PA, Thomson AJ, Ralph ET, Guest JR, Green J. FNR is a direct oxygen sensor having a biphasic response curve. FEBS Lett 1997; 416:349-52; PMID:9373183; http://dx.doi.org/10.1016/S0014-5793(97)01219-2 9373183

[15] Khoroshilova N, Popescu C, Münck E, Beinert H, Kiley PJ. Iron-sulfur cluster disassembly in the FNR protein of *Escherichia coli* by O_2_: [4Fe-4S] to [2Fe-2S] conversion with loss of biological activity. Proc Natl Acad Sci U S A 1997; 94:6087-92; PMID:9177174; http://dx.doi.org/10.1073/pnas.94.12.6087 9177174 PMC21006

[16] Popescu CV, Bates DM, Beinert H, Münck E, Kiley PJ. Mössbauer spectroscopy as a tool for the study of activation/inactivation of the transcription regulator FNR in whole cells of *Escherichia coli*. Proc Natl Acad Sci U S A 1998; 95:13431-5; PMID:9811817; http://dx.doi.org/10.1073/pnas.95.23.13431 9811817 PMC24836

[17] Becker S, Holighaus G, Gabrielczyk T, Unden G. O_2_ as the regulatory signal for FNR-dependent gene regulation in *Escherichia coli*. J Bacteriol 1996; 178:4515-21; PMID:8755879 8755879 10.1128/jb.178.15.4515-4521.1996 PMC178218

[18] Green J, Bennett B, Jordan P, Ralph ET, Thomson AJ, Guest JR. Reconstitution of the [4Fe-4S] cluster in FNR and demonstration of the aerobic-anaerobic transcription switch *in vitro*. Biochem J 1996; 316:887-92; PMID:8670167 8670167 10.1042/bj3160887 PMC1217433

[19] Lazazzera BA, Beinert H, Khoroshilova N, Kennedy MC, Kiley PJ. DNA binding and dimerization of the Fe-S-containing FNR protein from *Escherichia coli* are regulated by oxygen. J Biol Chem 1996; 271:2762-8; PMID:8576252; http://dx.doi.org/10.1074/jbc.271.5.2762 8576252

[20] Sutton VR, Stubna A, Patschkowski T, Münck E, Beinert H, Kiley PJ. Superoxide destroys the [2Fe-2S]^2+^ cluster of FNR from *Escherichia coli*. Biochemistry 2004; 43:791-8; PMID:14730984; http://dx.doi.org/10.1021/bi0357053 14730984

[21] Reinhart F, Achebach S, Koch T, Unden G. Reduced apo-fumarate nitrate reductase regulator (apoFNR) as the major form of FNR in aerobically growing *Escherichia coli.* J Bacteriol 2008; 190:879-86; PMID:18055593; http://dx.doi.org/10.1128/JB.01374-07 18055593 PMC2223584

[22] Achebach S, Selmer T, Unden G. Properties and significance of apoFNR as a second form of air-inactivated [4Fe-4S].FNR of *Escherichia coli*. FEBS J 2005; 272:4260-9; PMID:16098206; http://dx.doi.org/10.1111/j.1742-4658.2005.04840.x 16098206

[23] Engel P, Trageser M, Unden G. Reversible interconversion of the functional state of the gene regulator FNR from *Escherichia coli in vivo* by O_2_ and iron availability. Arch Microbiol 1991; 156:463-70; PMID:1785953 1785953 10.1007/BF00245393

[24] Mettert EL, Kiley PJ. ClpXP-dependent proteolysis of FNR upon loss of its O_2_-sensing [4Fe-4S] cluster. J Mol Biol 2005; 354:220-32; PMID:16243354; http://dx.doi.org/10.1016/j.jmb.2005.09.066 16243354

[25] Dibden DP, Green J. *In vivo* cycling of the *Escherichia coli* transcription factor FNR between active and inactive states. Microbiology 2005; 151:4063-70; PMID:16339951; http://dx.doi.org/10.1099/mic.0.28253-0 16339951

[26] Zhang B, Crack JC, Subramanian S, Green J, Thomson AJ, Le Brun NE, Johnson MK. Reversible cycling between cysteine persulfide-ligated [2Fe-2S] and cysteine-ligated [4Fe-4S] clusters in the FNR regulatory protein. Proc Natl Acad Sci U S A 2012; 109:15734-9; PMID:23019358; http://dx.doi.org/10.1073/pnas.1208787109 23019358 PMC3465412

[27] Jervis AJ, Crack JC, White G, Artymiuk PJ, Cheesman MR, Thomson AJ, Le Brun NE, Green J. The O_2_ sensitivity of the transcription factor FNR is controlled by Ser24 modulating the kinetics of [4Fe-4S] to [2Fe-2S] conversion. Proc Natl Acad Sci U S A 2009; 106:4659-64; PMID:19261852; http://dx.doi.org/10.1073/pnas.0804943106 19261852 PMC2660729

[28] Sutton VR, Mettert EL, Beinert H, Kiley PJ. Kinetic analysis of the oxidative conversion of the [4Fe-4S]^2+^ cluster of FNR to a [2Fe-2S]^2+^ Cluster. J Bacteriol 2004; 186:8018-25; PMID:15547274; http://dx.doi.org/10.1128/JB.186.23.8018-8025.2004 15547274 PMC529072

[29] Fink RC, Evans MR, Porwollik S, Vazquez-Torres A, Jones-Carson J, Troxell B, Libby SJ, McClelland M, Hassan HM. FNR is a global regulator of virulence and anaerobic metabolism in *Salmonella enterica* serovar Typhimurium (ATCC 14028s). J Bacteriol 2007; 189:2262-73; PMID:17220229; http://dx.doi.org/10.1128/JB.00726-06 17220229 PMC1899381

[30] Wood GE, Khelef N, Guiso N, Friedman RL. Identification of Btr-regulated genes using a titration assay. Search for a role for this transcriptional regulator in the growth and virulence of *Bordetella pertussis*. Gene 1998; 209:51-8; PMID:9583950; http://dx.doi.org/10.1016/S0378-1119(98)00031-6 9583950

[31] Bartolini E, Frigimelica E, Giovinazzi S, Galli G, Shaik Y, Genco C, Welsch JA, Granoff DM, Grandi G, Grifantini R. Role of FNR and FNR-regulated, sugar fermentation genes in *Neisseria meningitidis* infection. Mol Microbiol 2006; 60:963-72; PMID:16677307; http://dx.doi.org/10.1111/j.1365-2958.2006.05163.x 16677307 PMC2258229

[32] Jackson AA, Gross MJ, Daniels EF, Hampton TH, Hammond JH, Vallet-Gely I, Dove SL, Stanton BA, Hogan DA. Anr and its activation by PlcH activity in *Pseudomonas aeruginosa* host colonization and virulence. J Bacteriol 2013; 195:3093-104; PMID:23667230; http://dx.doi.org/10.1128/JB.02169-12 23667230 PMC3697539

[33] Kuntumalla S, Zhang Q, Braisted JC, Fleischmann RD, Peterson SN, Donohue-Rolfe A, Tzipori S, Pieper R. *In vivo* versus *in vitro* protein abundance analysis of *Shigella dysenteriae* type 1 reveals changes in the expression of proteins involved in virulence, stress and energy metabolism. BMC Microbiol 2011; 11:147; PMID:21702961; http://dx.doi.org/10.1186/1471-2180-11-147 21702961 PMC3136414

[34] Lai XH, Arencibia I, Johansson A, Wai SN, Oscarsson J, Kalfas S, Sundqvist KG, Mizunoe Y, Sjöstedt A, Uhlin BE. Cytocidal and apoptotic effects of the ClyA protein from *Escherichia coli* on primary and cultured monocytes and macrophages. Infect Immun 2000; 68:4363-7; PMID:10858262; http://dx.doi.org/10.1128/IAI.68.7.4363-4367.2000 10858262 PMC101772

[35] Mueller M, Grauschopf U, Maier T, Glockshuber R, Ban N. The structure of a cytolytic α-helical toxin pore reveals its assembly mechanism. Nature 2009; 459:726-30; PMID:19421192; http://dx.doi.org/10.1038/nature08026 19421192

[36] Ong EB, Anthony AA, Ismail A, Lim TS Cloning, expression, and purification of the hemolysin/cytolysin (HlyE antigen) from *Salmonella enterica* serovar Typhi: potential application for immunoassay development. Diagn Microbiol Infect Dis 2013 10.1016/j.diagmicrobio.2013.05.010 23790417

[37] Suez J, Porwollik S, Dagan A, Marzel A, Schorr YI, Desai PT, Agmon V, McClelland M, Rahav G, Gal-Mor O. Virulence gene profiling and pathogenicity characterization of non-typhoidal *Salmonella* accounted for invasive disease in humans. PLoS One 2013; 8:e58449; PMID:23505508; http://dx.doi.org/10.1371/journal.pone.0058449 23505508 PMC3591323

[38] von Rhein C, Bauer S, López Sanjurjo EJ, Benz R, Goebel W, Ludwig A. ClyA cytolysin from *Salmonella*: distribution within the genus, regulation of expression by SlyA, and pore-forming characteristics. Int J Med Microbiol 2009; 299:21-35; PMID:18715828; http://dx.doi.org/10.1016/j.ijmm.2008.06.004 18715828

[39] Wallace AJ, Stillman TJ, Atkins A, Jamieson SJ, Bullough PA, Green J, Artymiuk PJ. *E. coli* hemolysin E (HlyE, ClyA, SheA): X-ray crystal structure of the toxin and observation of membrane pores by electron microscopy. Cell 2000; 100:265-76; PMID:10660049; http://dx.doi.org/10.1016/S0092-8674(00)81564-0 10660049

[40] Wyborn NR, Clark A, Roberts RE, Jamieson SJ, Tzokov S, Bullough PA, Stillman TJ, Artymiuk PJ, Galen JE, Zhao L, et al. Properties of haemolysin E (HlyE) from a pathogenic *Escherichia coli* avian isolate and studies of HlyE export. Microbiology 2004; 150:1495-505; PMID:15133111; http://dx.doi.org/10.1099/mic.0.26877-0 15133111

[41] Green J, Baldwin ML. The molecular basis for the differential regulation of the *hlyE*-encoded haemolysin of *Escherichia coli* by FNR and HlyX lies in the improved activating region 1 contact of HlyX. Microbiology 1997; 143:3785-93; PMID:9421903; http://dx.doi.org/10.1099/00221287-143-12-3785 9421903

[42] Lithgow JK, Haider F, Roberts IS, Green J. Alternate SlyA and H-NS nucleoprotein complexes control *hlyE* expression in *Escherichia coli* K-12. Mol Microbiol 2007; 66:685-98; PMID:17892462; http://dx.doi.org/10.1111/j.1365-2958.2007.05950.x 17892462 PMC2156107

[43] Murase K, Ooka T, Iguchi A, Ogura Y, Nakayama K, Asadulghani M, Islam MR, Hiyoshi H, Kodama T, Beutin L, et al. Haemolysin E- and enterohaemolysin-derived haemolytic activity of O55/O157 strains and other *Escherichia coli* lineages. Microbiology 2012; 158:746-58; PMID:22194351; http://dx.doi.org/10.1099/mic.0.054775-0 22194351

[44] Westermark M, Oscarsson J, Mizunoe Y, Urbonaviciene J, Uhlin BE. Silencing and activation of ClyA cytotoxin expression in *Escherichia coli*. J Bacteriol 2000; 182:6347-57; PMID:11053378; http://dx.doi.org/10.1128/JB.182.22.6347-6357.2000 11053378 PMC94780

[45] Wyborn NR, Stapleton MR, Norte VA, Roberts RE, Grafton J, Green J. Regulation of *Escherichia coli* hemolysin E expression by H-NS and *Salmonella* SlyA. J Bacteriol 2004; 186:1620-8; PMID:14996792; http://dx.doi.org/10.1128/JB.186.6.1620-1628.2004 14996792 PMC355951

[46] Fuentes JA, Villagra N, Castillo-Ruiz M, Mora GC. The *Salmonella* Typhi *hlyE* gene plays a role in invasion of cultured epithelial cells and its functional transfer to *S*. Typhimurium promotes deep organ infection in mice. Res Microbiol 2008; 159:279-87; PMID:18434098; http://dx.doi.org/10.1016/j.resmic.2008.02.006 18434098

[47] Hunt S, Green J, Artymiuk PJ. Hemolysin E (HlyE, ClyA, SheA) and related toxins. Adv Exp Med Biol 2010; 677:116-26; PMID:20687485; http://dx.doi.org/10.1007/978-1-4419-6327-7_10 20687485

[48] Esbelin J, Armengaud J, Zigha A, Duport C. ResDE-dependent regulation of enterotoxin gene expression in *Bacillus cereus*: evidence for multiple modes of binding for ResD and interaction with Fnr. J Bacteriol 2009; 191:4419-26; PMID:19395489; http://dx.doi.org/10.1128/JB.00321-09 19395489 PMC2698483

[49] Esbelin J, Jouanneau Y, Armengaud J, Duport C. ApoFnr binds as a monomer to promoters regulating the expression of enterotoxin genes of *Bacillus cereus*. J Bacteriol 2008; 190:4242-51; PMID:18424517; http://dx.doi.org/10.1128/JB.00336-08 18424517 PMC2446755

[50] Esbelin J, Jouanneau Y, Duport C. *Bacillus cereus* Fnr binds a [4Fe-4S] cluster and forms a ternary complex with ResD and PlcR. BMC Microbiol 2012; 12:125; PMID:22731107; http://dx.doi.org/10.1186/1471-2180-12-125 22731107 PMC3520743

[51] Marteyn B, West NP, Browning DF, Cole JA, Shaw JG, Palm F, Mounier J, Prévost MC, Sansonetti P, Tang CM. Modulation of *Shigella* virulence in response to available oxygen *in vivo*. Nature 2010; 465:355-8; PMID:20436458; http://dx.doi.org/10.1038/nature08970 20436458 PMC3750455

[52] O’Callaghan J, Reen FJ, Adams C, Casey PG, Gahan CG, O’Gara F. A novel host-responsive sensor mediates virulence and type III secretion during *Pseudomonas aeruginosa*-host cell interactions. Microbiology 2012; 158:1057-70; PMID:22262100; http://dx.doi.org/10.1099/mic.0.056127-0 22262100

[53] Bekker M, Alexeeva S, Laan W, Sawers G, Teixeira de Mattos J, Hellingwerf K. The ArcBA two-component system of *Escherichia coli* is regulated by the redox state of both the ubiquinone and the menaquinone pool. J Bacteriol 2010; 192:746-54; PMID:19933363; http://dx.doi.org/10.1128/JB.01156-09 19933363 PMC2812447

[54] Georgellis D, Kwon O, Lin EC. Quinones as the redox signal for the *arc* two-component system of bacteria. Science 2001; 292:2314-6; PMID:11423658; http://dx.doi.org/10.1126/science.1059361 11423658

[55] Malpica R, Franco B, Rodriguez C, Kwon O, Georgellis D. Identification of a quinone-sensitive redox switch in the ArcB sensor kinase. Proc Natl Acad Sci U S A 2004; 101:13318-23; PMID:15326287; http://dx.doi.org/10.1073/pnas.0403064101 15326287 PMC516565

[56] Boll EJ, Nielsen LN, Krogfelt KA, Struve C. Novel screening assay for *in vivo* selection of *Klebsiella pneumoniae* genes promoting gastrointestinal colonisation. BMC Microbiol 2012; 12:201; PMID:22967317; http://dx.doi.org/10.1186/1471-2180-12-201 22967317 PMC3463446

[57] Boulette ML, Payne SM. Anaerobic regulation of *Shigella flexneri* virulence: ArcA regulates Fur and iron acquisition genes. J Bacteriol 2007; 189:6957-67; PMID:17660284; http://dx.doi.org/10.1128/JB.00621-07 17660284 PMC2045222

[58] Lu S, Killoran PB, Fang FC, Riley LW. The global regulator ArcA controls resistance to reactive nitrogen and oxygen intermediates in Salmonella enterica serovar Enteritidis. Infect Immun 2002; 70:451-61; PMID:11796570; http://dx.doi.org/10.1128/IAI.70.2.451-461.2002 11796570 PMC127680

[59] Serna A, Espinosa E, Camacho EM, Casadesús J. Regulation of bacterial conjugation in microaerobiosis by host-encoded functions ArcAB and *sdhABCD*. Genetics 2010; 184:947-58; PMID:20083612; http://dx.doi.org/10.1534/genetics.109.109918 20083612 PMC2865929

[60] Wong SM, Akerley BJ. Genome-scale approaches to identify genes essential for *Haemophilus influenzae* pathogenesis. Front Cell Infect Microbiol 2012; 2:23; PMID:22919615; http://dx.doi.org/10.3389/fcimb.2012.00023 22919615 PMC3417392

[61] Wong SM, St Michael F, Cox A, Ram S, Akerley BJ. ArcA-regulated glycosyltransferase lic2B promotes complement evasion and pathogenesis of nontypeable *Haemophilus influenzae*. Infect Immun 2011; 79:1971-83; PMID:21357723; http://dx.doi.org/10.1128/IAI.01269-10 21357723 PMC3088160

[62] Sengupta N, Paul K, Chowdhury R. The global regulator ArcA modulates expression of virulence factors in *Vibrio cholerae*. Infect Immun 2003; 71:5583-9; PMID:14500477; http://dx.doi.org/10.1128/IAI.71.10.5583-5589.2003 14500477 PMC201065

[63] Buettner FF, Maas A, Gerlach GF. An *Actinobacillus pleuropneumoniae arcA* deletion mutant is attenuated and deficient in biofilm formation. Vet Microbiol 2008; 127:106-15; PMID:17881160; http://dx.doi.org/10.1016/j.vetmic.2007.08.005 17881160

[64] Stern AM, Liu B, Bakken LR, Shapleigh JP, Zhu J. A novel protein protects bacterial iron-dependent metabolism from nitric oxide. J Bacteriol 2013; 195:4702-8; PMID:23935055; http://dx.doi.org/10.1128/JB.00836-13 23935055 PMC3807435

[65] Hausladen A, Gow A, Stamler JS. Flavohemoglobin denitrosylase catalyzes the reaction of a nitroxyl equivalent with molecular oxygen. Proc Natl Acad Sci U S A 2001; 98:10108-12; PMID:11517313; http://dx.doi.org/10.1073/pnas.181199698 11517313 PMC56923

[66] Gilberthorpe NJ, Lee ME, Stevanin TM, Read RC, Poole RK. NsrR: a key regulator circumventing *Salmonella enterica* serovar Typhimurium oxidative and nitrosative stress *in vitro* and in IFN-gamma-stimulated J774.2 macrophages. Microbiology 2007; 153:1756-71; PMID:17526833; http://dx.doi.org/10.1099/mic.0.2006/003731-0 17526833 PMC2884951

[67] Bang IS, Liu L, Vazquez-Torres A, Crouch ML, Stamler JS, Fang FC. Maintenance of nitric oxide and redox homeostasis by the *salmonella* flavohemoglobin hmp. J Biol Chem 2006; 281:28039-47; PMID:16873371; http://dx.doi.org/10.1074/jbc.M605174200 16873371

[68] Svensson L, Poljakovic M, Säve S, Gilberthorpe N, Schön T, Strid S, Corker H, Poole RK, Persson K. Role of flavohemoglobin in combating nitrosative stress in uropathogenic *Escherichia coli*–implications for urinary tract infection. Microb Pathog 2010; 49:59-66; PMID:20399845; http://dx.doi.org/10.1016/j.micpath.2010.04.001 20399845

[69] Arai H, Iiyama K. Role of nitric oxide-detoxifying enzymes in the virulence of *Pseudomonas aeruginosa* against the silkworm, *Bombyx mori*. Biosci Biotechnol Biochem 2013; 77:198-200; PMID:23291757; http://dx.doi.org/10.1271/bbb.120656 23291757

[70] Vinogradov SN, Tinajero-Trejo M, Poole RK, Hoogewijs D. Bacterial and archaeal globins - a revised perspective. Biochim Biophys Acta 2013; 1834:1789-800; PMID:23541529; http://dx.doi.org/10.1016/j.bbapap.2013.03.021 23541529

[71] Tinajero-Trejo M, Vreugdenhil A, Sedelnikova SE, Davidge KS, Poole RK. Nitric oxide reactivities of the two globins of the foodborne pathogen Campylobacter jejuni: roles in protection from nitrosative stress and analysis of potential reductants. Nitric Oxide 2013; 34:65-75; PMID:23764490; http://dx.doi.org/10.1016/j.niox.2013.06.002 23764490

[72] Elvers KT, Wu G, Gilberthorpe NJ, Poole RK, Park SF. Role of an inducible single-domain hemoglobin in mediating resistance to nitric oxide and nitrosative stress in *Campylobacter jejuni* and *Campylobacter coli*. J Bacteriol 2004; 186:5332-41; PMID:15292134; http://dx.doi.org/10.1128/JB.186.16.5332-5341.2004 15292134 PMC490904

[73] Elvers KT, Turner SM, Wainwright LM, Marsden G, Hinds J, Cole JA, Poole RK, Penn CW, Park SF. NssR, a member of the Crp-Fnr superfamily from *Campylobacter jejuni*, regulates a nitrosative stress-responsive regulon that includes both a single-domain and a truncated haemoglobin. Mol Microbiol 2005; 57:735-50; PMID:16045618; http://dx.doi.org/10.1111/j.1365-2958.2005.04723.x 16045618

[74] Gardner AM, Helmick RA, Gardner PR. Flavorubredoxin, an inducible catalyst for nitric oxide reduction and detoxification in *Escherichia coli*. J Biol Chem 2002; 277:8172-7; PMID:11751865; http://dx.doi.org/10.1074/jbc.M110471200 11751865

[75] Shimizu T, Tsutsuki H, Matsumoto A, Nakaya H, Noda M. The nitric oxide reductase of enterohaemorrhagic *Escherichia coli* plays an important role for the survival within macrophages. Mol Microbiol 2012; 85:492-512; PMID:22716767; http://dx.doi.org/10.1111/j.1365-2958.2012.08122.x 22716767

[76] Justino MC, Ecobichon C, Fernandes AF, Boneca IG, Saraiva LM. *Helicobacter pylori* has an unprecedented nitric oxide detoxifying system. Antioxid Redox Signal 2012; 17:1190-200; PMID:22236381; http://dx.doi.org/10.1089/ars.2011.4304 22236381

[77] Bueno E, Mesa S, Bedmar EJ, Richardson DJ, Delgado MJ. Bacterial adaptation of respiration from oxic to microoxic and anoxic conditions: redox control. Antioxid Redox Signal 2012; 16:819-52; PMID:22098259; http://dx.doi.org/10.1089/ars.2011.4051 22098259 PMC3283443

[78] van Wonderen JH, Burlat B, Richardson DJ, Cheesman MR, Butt JN. The nitric oxide reductase activity of cytochrome *c* nitrite reductase from *Escherichia coli*. J Biol Chem 2008; 283:9587-94; PMID:18245085; http://dx.doi.org/10.1074/jbc.M709090200 18245085

[79] Mills PC, Rowley G, Spiro S, Hinton JC, Richardson DJ. A combination of cytochrome *c* nitrite reductase (NrfA) and flavorubredoxin (NorV) protects *Salmonella enterica* serovar Typhimurium against killing by NO in anoxic environments. Microbiology 2008; 154:1218-28; PMID:18375814; http://dx.doi.org/10.1099/mic.0.2007/014290-0 18375814

[80] Vine CE, Cole JA. Unresolved sources, sinks, and pathways for the recovery of enteric bacteria from nitrosative stress. FEMS Microbiol Lett 2011; 325:99-107; PMID:22029434; http://dx.doi.org/10.1111/j.1574-6968.2011.02425.x 22029434

[81] Rodionov DA, Dubchak IL, Arkin AP, Alm EJ, Gelfand MS. Dissimilatory metabolism of nitrogen oxides in bacteria: comparative reconstruction of transcriptional networks. PLoS Comput Biol 2005; 1:e55; PMID:16261196; http://dx.doi.org/10.1371/journal.pcbi.0010055 16261196 PMC1274295

[82] Tucker NP, Le Brun NE, Dixon R, Hutchings MI. There's NO stopping NsrR, a global regulator of the bacterial NO stress response. Trends Microbiol 2010; 18:149-56; PMID:20167493; http://dx.doi.org/10.1016/j.tim.2009.12.009 20167493

[83] Partridge JD, Bodenmiller DM, Humphrys MS, Spiro S. NsrR targets in the *Escherichia coli* genome: new insights into DNA sequence requirements for binding and a role for NsrR in the regulation of motility. Mol Microbiol 2009; 73:680-94; PMID:19656291; http://dx.doi.org/10.1111/j.1365-2958.2009.06799.x 19656291

[84] Karlinsey JE, Bang IS, Becker LA, Frawley ER, Porwollik S, Robbins HF, Thomas VC, Urbano R, McClelland M, Fang FC. The NsrR regulon in nitrosative stress resistance of *Salmonella enterica* serovar Typhimurium. Mol Microbiol 2012; 85:1179-93; PMID:22831173; http://dx.doi.org/10.1111/j.1365-2958.2012.08167.x 22831173 PMC3438343

[85] Isabella VM, Lapek JD Jr., Kennedy EM, Clark VL. Functional analysis of NsrR, a nitric oxide-sensing Rrf2 repressor in *Neisseria gonorrhoeae*. Mol Microbiol 2009; 71:227-39; PMID:19007408; http://dx.doi.org/10.1111/j.1365-2958.2008.06522.x 19007408 PMC2630374

[86] Tucker NP, Hicks MG, Clarke TA, Crack JC, Chandra G, Le Brun NE, Dixon R, Hutchings MI. The transcriptional repressor protein NsrR senses nitric oxide directly via a [2Fe-2S] cluster. PLoS One 2008; 3:e3623; PMID:18989365; http://dx.doi.org/10.1371/journal.pone.0003623 18989365 PMC2577008

[87] Yukl ET, Elbaz MA, Nakano MM, Moënne-Loccoz P. Transcription factor NsrR from *Bacillus subtilis* senses nitric oxide with a 4Fe-4S cluster. Biochemistry 2008; 47:13084-92; PMID:19006327; http://dx.doi.org/10.1021/bi801342x 19006327 PMC2891187

[88] Kommineni S, Yukl E, Hayashi T, Delepine J, Geng H, Moënne-Loccoz P, Nakano MM. Nitric oxide-sensitive and -insensitive interaction of *Bacillus subtilis* NsrR with a ResDE-controlled promoter. Mol Microbiol 2010; 78:1280-93; PMID:21091510; http://dx.doi.org/10.1111/j.1365-2958.2010.07407.x 21091510 PMC3075490

[89] D’Autréaux B, Tucker NP, Dixon R, Spiro S. A non-haem iron centre in the transcription factor NorR senses nitric oxide. Nature 2005; 437:769-72; PMID:16193057; http://dx.doi.org/10.1038/nature03953 16193057

[90] Tucker NP, D’Autréaux B, Yousafzai FK, Fairhurst SA, Spiro S, Dixon R. Analysis of the nitric oxide-sensing non-heme iron center in the NorR regulatory protein. J Biol Chem 2008; 283:908-18; PMID:18003617; http://dx.doi.org/10.1074/jbc.M705850200 18003617

[91] Bush M, Ghosh T, Tucker N, Zhang X, Dixon R. Nitric oxide-responsive interdomain regulation targets the σ^54^-interaction surface in the enhancer binding protein NorR. Mol Microbiol 2010; 77:1278-88; PMID:20624215; http://dx.doi.org/10.1111/j.1365-2958.2010.07290.x 20624215 PMC2941729

[92] Tucker NP, Ghosh T, Bush M, Zhang X, Dixon R. Essential roles of three enhancer sites in sigma54-dependent transcription by the nitric oxide sensing regulatory protein NorR. Nucleic Acids Res 2010; 38:1182-94; PMID:19955233; http://dx.doi.org/10.1093/nar/gkp1065 19955233 PMC2831303

[93] Stern AM, Hay AJ, Liu Z, Desland FA, Zhang J, Zhong Z, Zhu J. The NorR regulon is critical for *Vibrio cholerae* resistance to nitric oxide and sustained colonization of the intestines. MBio 2012; 3:e00013-12; PMID:22511349; http://dx.doi.org/10.1128/mBio.00013-12 22511349 PMC3345576

[94] Smith HK, Shepherd M, Monk C, Green J, Poole RK. The NO-responsive hemoglobins of *Campylobacter jejuni*: concerted responses of two globins to NO and evidence *in vitro* for globin regulation by the transcription factor NssR. Nitric Oxide 2011; 25:234-41; PMID:21199674; http://dx.doi.org/10.1016/j.niox.2010.12.009 21199674

[95] Cruz-Ramos H, Crack J, Wu G, Hughes MN, Scott C, Thomson AJ, Green J, Poole RK. NO sensing by FNR: regulation of the *Escherichia coli* NO-detoxifying flavohaemoglobin, Hmp. EMBO J 2002; 21:3235-44; PMID:12093725; http://dx.doi.org/10.1093/emboj/cdf339 12093725 PMC126088

[96] Crack JC, Le Brun NE, Thomson AJ, Green J, Jervis AJ. Reactions of nitric oxide and oxygen with the regulator of fumarate and nitrate reduction, a global transcriptional regulator, during anaerobic growth of *Escherichia coli*. Methods Enzymol 2008; 437:191-209; PMID:18433630; http://dx.doi.org/10.1016/S0076-6879(07)37011-0 18433630

[97] Crack JC, Stapleton MR, Green J, Thomson AJ, Le Brun NE. Mechanism of [4Fe-4S](Cys)_4_ cluster nitrosylation is conserved among NO-responsive regulators. J Biol Chem 2013; 288:11492-502; PMID:23471974; http://dx.doi.org/10.1074/jbc.M112.439901 23471974 PMC3630887

[98] Pomposiello PJ, Bennik MH, Demple B. Genome-wide transcriptional profiling of the *Escherichia coli* responses to superoxide stress and sodium salicylate. J Bacteriol 2001; 183:3890-902; PMID:11395452; http://dx.doi.org/10.1128/JB.183.13.3890-3902. 2001 11395452 PMC95271

[99] Gu M, Imlay JA. The SoxRS response of *Escherichia coli* is directly activated by redox-cycling drugs rather than by superoxide. Mol Microbiol 2011; 79:1136-50; PMID:21226770; http://dx.doi.org/10.1111/j.1365-2958.2010.07520.x 21226770 PMC3071027

[100] Imlay J, Gu M. Many plants and bacteria excrete redox-cycling compounds. Free Radic Biol Med 2011; 50:1814-5; PMID:21466847; http://dx.doi.org/10.1016/j.freeradbiomed.2011.03.034 21466847

[101] Liochev SI, Fridovich I. Is superoxide able to induce SoxRS? Free Radic Biol Med 2011; 50:1813; PMID:21459140; http://dx.doi.org/10.1016/j.freeradbiomed.2011.03.029 21459140

[102] Dietrich LE, Teal TK, Price-Whelan A, Newman DK. Redox-active antibiotics control gene expression and community behavior in divergent bacteria. Science 2008; 321:1203-6; PMID:18755976; http://dx.doi.org/10.1126/science.1160619 18755976 PMC2745639

[103] Piddock LJ. Fluoroquinolone resistance in *Salmonella* serovars isolated from humans and food animals. FEMS Microbiol Rev 2002; 26:3-16; PMID:12007640 12007640 10.1111/j.1574-6976.2002.tb00596.x

[104] Ha U, Jin S. Expression of the *soxR* gene of *Pseudomonas aeruginosa* is inducible during infection of burn wounds in mice and is required to cause efficient bacteremia. Infect Immun 1999; 67:5324-31; PMID:10496912 10496912 10.1128/iai.67.10.5324-5331.1999 PMC96887

[105] Palma M, Zurita J, Ferreras JA, Worgall S, Larone DH, Shi L, Campagne F, Quadri LE. *Pseudomonas aeruginosa* SoxR does not conform to the archetypal paradigm for SoxR-dependent regulation of the bacterial oxidative stress adaptive response. Infect Immun 2005; 73:2958-66; PMID:15845502; http://dx.doi.org/10.1128/IAI.73.5.2958-2966. 2005 15845502 PMC1087365

[106] Kang IH, Kim JS, Lee JK. The virulence of *Vibrio vulnificus* is affected by the cellular level of superoxide dismutase activity. J Microbiol Biotechnol 2007; 17:1399-402; PMID:18051612 18051612

[107] Mahavihakanont A, Charoenlap N, Namchaiw P, Eiamphungporn W, Chattrakarn S, Vattanaviboon P, Mongkolsuk S. Novel roles of SoxR, a transcriptional regulator from *Xanthomonas campestris*, in sensing redox-cycling drugs and regulating a protective gene that have overall implications for bacterial stress physiology and virulence on a host plant. J Bacteriol 2012; 194:209-17; PMID:22056938; http://dx.doi.org/10.1128/JB.05603-11 22056938 PMC3256661

[108] Watanabe S, Kita A, Kobayashi K, Miki K. Crystal structure of the [2Fe-2S] oxidative-stress sensor SoxR bound to DNA. Proc Natl Acad Sci U S A 2008; 105:4121-6; PMID:18334645; http://dx.doi.org/10.1073/pnas.0709188105 18334645 PMC2393793

[109] Gorodetsky AA, Dietrich LE, Lee PE, Demple B, Newman DK, Barton JK. DNA binding shifts the redox potential of the transcription factor SoxR. Proc Natl Acad Sci U S A 2008; 105:3684-9; PMID:18316718; http://dx.doi.org/10.1073/pnas.0800093105 18316718 PMC2268809

[110] Koo MS, Lee JH, Rah SY, Yeo WS, Lee JW, Lee KL, Koh YS, Kang SO, Roe JH. A reducing system of the superoxide sensor SoxR in *Escherichia coli*. EMBO J 2003; 22:2614-22; PMID:12773378; http://dx.doi.org/10.1093/emboj/cdg252 12773378 PMC156749

[111] Griffith KL, Shah IM, Wolf RE Jr. Proteolytic degradation of *Escherichia coli* transcription activators SoxS and MarA as the mechanism for reversing the induction of the superoxide (SoxRS) and multiple antibiotic resistance (Mar) regulons. Mol Microbiol 2004; 51:1801-16; PMID:15009903; http://dx.doi.org/10.1046/j.1365-2958.2003.03952.x 15009903

[112] Nunoshiba T, deRojas-Walker T, Wishnok JS, Tannenbaum SR, Demple B. Activation by nitric oxide of an oxidative-stress response that defends *Escherichia coli* against activated macrophages. Proc Natl Acad Sci U S A 1993; 90:9993-7; PMID:8234347; http://dx.doi.org/10.1073/pnas.90.21.9993 8234347 PMC47699

[113] Ding H, Demple B. Direct nitric oxide signal transduction via nitrosylation of iron-sulfur centers in the SoxR transcription activator. Proc Natl Acad Sci U S A 2000; 97:5146-50; PMID:10805777; http://dx.doi.org/10.1073/pnas.97.10.5146 10805777 PMC25796

[114] Pullan ST, Gidley MD, Jones RA, Barrett J, Stevanin TM, Read RC, Green J, Poole RK. Nitric oxide in chemostat-cultured Escherichia coli is sensed by Fnr and other global regulators: unaltered methionine biosynthesis indicates lack of *S* nitrosation. J Bacteriol 2007; 189:1845-55; PMID:17189370; http://dx.doi.org/10.1128/JB.01354-06 17189370 PMC1855760

[115] Vasil’eva SV, Stupakova MV, Lobysheva II, Mikoyan VD, Vanin AF. Activation of the *Escherichia coli* SoxRS-regulon by nitric oxide and its physiological donors. Biochemistry (Mosc) 2001; 66:984-8; PMID:11703180; http://dx.doi.org/10.1023/A:1012317508971 11703180

[116] Zheng M, Wang X, Templeton LJ, Smulski DR, LaRossa RA, Storz G. DNA microarray-mediated transcriptional profiling of the *Escherichia coli* response to hydrogen peroxide. J Bacteriol 2001; 183:4562-70; PMID:11443091; http://dx.doi.org/10.1128/JB.183.15.4562-4570.2001 11443091 PMC95351

[117] Choi H, Kim S, Mukhopadhyay P, Cho S, Woo J, Storz G, Ryu SE. Structural basis of the redox switch in the OxyR transcription factor. Cell 2001; 105:103-13; PMID:11301006; http://dx.doi.org/10.1016/S0092-8674(01)00300-2 11301006

[118] Kim SO, Merchant K, Nudelman R, Beyer WF Jr., Keng T, DeAngelo J, Hausladen A, Stamler JS. OxyR: a molecular code for redox-related signaling. Cell 2002; 109:383-96; PMID:12015987; http://dx.doi.org/10.1016/S0092-8674(02)00723-7 12015987

[119] Charoenlap N, Buranajitpakorn S, Duang-Nkern J, Namchaiw P, Vattanaviboon P, Mongkolsuk S. Evaluation of the virulence of *Xanthomonas campestris* pv. campestris mutant strains lacking functional genes in the OxyR regulon. Curr Microbiol 2011; 63:232-7; PMID:21710133; http://dx.doi.org/10.1007/s00284-011-9970-9 21710133

[120] Erickson DL, Russell CW, Johnson KL, Hileman T, Stewart RM. PhoP and OxyR transcriptional regulators contribute to Yersinia pestis virulence and survival within Galleria mellonella. Microb Pathog 2011; 51:389-95; PMID:21964409; http://dx.doi.org/10.1016/j.micpath.2011.08.008 21964409

[121] Flores-Cruz Z, Allen C. Necessity of OxyR for the hydrogen peroxide stress response and full virulence in *Ralstonia solanacearum*. Appl Environ Microbiol 2011; 77:6426-32; PMID:21803891; http://dx.doi.org/10.1128/AEM.05813-11 21803891 PMC3187169

[122] Hennequin C, Forestier C. *oxyR*, a LysR-type regulator involved *in Klebsiella pneumoniae* mucosal and abiotic colonization. Infect Immun 2009; 77:5449-57; PMID:19786563; http://dx.doi.org/10.1128/IAI.00837-09 19786563 PMC2786449

[123] Johnson JR, Clabots C, Rosen H. Effect of inactivation of the global oxidative stress regulator *oxyR* on the colonization ability of *Escherichia coli* O1:K1:H7 in a mouse model of ascending urinary tract infection. Infect Immun 2006; 74:461-8; PMID:16369002; http://dx.doi.org/10.1128/IAI.74.1.461-468.2006 16369002 PMC1346679

[124] Johnson JR, Russo TA, Drawz SM, Clabots C, Olson R, Kuskowski MA, Rosen H. OxyR contributes to the virulence of a Clonal Group A *Escherichia coli* strain (O17:K^+^:H18) in animal models of urinary tract infection, subcutaneous infection, and systemic sepsis. Microb Pathog 2013; 64:1-5; PMID:23850958; http://dx.doi.org/10.1016/j.micpath.2013.07.001 23850958

[125] Lau GW, Britigan BE, Hassett DJ. *Pseudomonas aeruginosa* OxyR is required for full virulence in rodent and insect models of infection and for resistance to human neutrophils. Infect Immun 2005; 73:2550-3; PMID:15784603; http://dx.doi.org/10.1128/IAI.73.4.2550-2553.2005 15784603 PMC1087439

[126] Moule MG, Monack DM, Schneider DS. Reciprocal analysis of *Francisella novicida* infections of a *Drosophila melanogaster* model reveal host-pathogen conflicts mediated by reactive oxygen and imd-regulated innate immune response. PLoS Pathog 2010; 6:e1001065; PMID:20865166; http://dx.doi.org/10.1371/journal.ppat.1001065 20865166 PMC2928790

[127] Pagán-Ramos E, Master SS, Pritchett CL, Reimschuessel R, Trucksis M, Timmins GS, Deretic V. Molecular and physiological effects of mycobacterial *oxyR* inactivation. J Bacteriol 2006; 188:2674-80; PMID:16547055; http://dx.doi.org/10.1128/JB.188.7.2674-2680.2006 16547055 PMC1428386

[128] Sund CJ, Rocha ER, Tzianabos AO, Wells WG, Gee JM, Reott MA, O’Rourke DP, Smith CJ. The *Bacteroides fragilis* transcriptome response to oxygen and H_2_O_2_: the role of OxyR and its effect on survival and virulence. Mol Microbiol 2008; 67:129-42; PMID:18047569; http://dx.doi.org/10.1111/j.1365-2958.2007.06031.x 18047569

[129] Rychlik I, Barrow PA. *Salmonella* stress management and its relevance to behaviour during intestinal colonisation and infection. FEMS Microbiol Rev 2005; 29:1021-40; PMID:16023758; http://dx.doi.org/10.1016/j.femsre.2005.03.005 16023758

[130] Hausladen A, Privalle CT, Keng T, DeAngelo J, Stamler JS. Nitrosative stress: activation of the transcription factor OxyR. Cell 1996; 86:719-29; PMID:8797819; http://dx.doi.org/10.1016/S0092-8674(00)80147-6 8797819

[131] Seth D, Hausladen A, Wang YJ, Stamler JS. Endogenous protein S-Nitrosylation in *E. coli*: regulation by OxyR. Science 2012; 336:470-3; PMID:22539721; http://dx.doi.org/10.1126/science.1215643 22539721 PMC3837355

[132] Lowy FD. *Staphylococcus aureus* infections. N Engl J Med 1998; 339:520-32; PMID:9709046; http://dx.doi.org/10.1056/NEJM199808203390806 9709046

[133] Härtig E, Jahn D. Regulation of the anaerobic metabolism in *Bacillus subtilis*. Adv Microb Physiol 2012; 61:195-216; PMID:23046954; http://dx.doi.org/10.1016/B978-0-12-394423-8.00005-6 23046954

[134] Novick RP, Geisinger E. Quorum sensing in staphylococci. Annu Rev Genet 2008; 541-64; PMID:18713030; http://dx.doi.org/10.1146/annurev.genet.42.110807.091640 18713030

[135] Pragman AA, Yarwood JM, Tripp TJ, Schlievert PM. Characterization of virulence factor regulation by SrrAB, a two-component system in *Staphylococcus aureus*. J Bacteriol 2004; 186:2430-8; PMID:15060046; http://dx.doi.org/10.1128/JB.186.8.2430-2438.2004 15060046 PMC412142

[136] Ulrich M, Bastian M, Cramton SE, Ziegler K, Pragman AA, Bragonzi A, Memmi G, Wolz C, Schlievert PM, Cheung A, et al. The staphylococcal respiratory response regulator SrrAB induces ica gene transcription and polysaccharide intercellular adhesin expression, protecting Staphylococcus aureus from neutrophil killing under anaerobic growth conditions. Mol Microbiol 2007; 65:1276-87; PMID:17697253; http://dx.doi.org/10.1111/j.1365-2958.2007.05863.x 17697253

[137] Fedtke I, Kamps A, Krismer B, Götz F. The nitrate reductase and nitrite reductase operons and the *narT* gene of *Staphylococcus carnosus* are positively controlled by the novel two-component system NreBC. J Bacteriol 2002; 184:6624-34; PMID:12426351; http://dx.doi.org/10.1128/JB.184.23.6624-6634.2002 12426351 PMC135434

[138] Kamps A, Achebach S, Fedtke I, Unden G, Götz F. Staphylococcal NreB: an O(_2_)-sensing histidine protein kinase with an O(_2_)-labile iron-sulphur cluster of the FNR type. Mol Microbiol 2004; 52:713-23; PMID:15101978; http://dx.doi.org/10.1111/j.1365-2958.2004.04024.x 15101978

[139] Müllner M, Hammel O, Mienert B, Schlag S, Bill E, Unden G. A PAS domain with an oxygen labile [4Fe-4S](^2+^) cluster in the oxygen sensor kinase NreB of *Staphylococcus carnosus*. Biochemistry 2008; 13921-32; PMID:19102705; http://dx.doi.org/10.1021/bi8014086 19102705

[140] Schlag S, Fuchs S, Nerz C, Gaupp R, Engelmann S, Liebeke M, Lalk M, Hecker M, Götz F. Characterization of the oxygen-responsive NreABC regulon of *Staphylococcus aureus*. J Bacteriol 2008; 190:7847-58; PMID:18820014; http://dx.doi.org/10.1128/JB.00905-08 18820014 PMC2583599

[141] Benton BM, Zhang JP, Bond S, Pope C, Christian T, Lee L, Winterberg KM, Schmid MB, Buysse JM. Large-scale identification of genes required for full virulence of *Staphylococcus aureus*. J Bacteriol 2004; 186:8478-89; PMID:15576798; http://dx.doi.org/10.1128/JB.186.24.8478-8489.2004 15576798 PMC532413

[142] Moormeier DE, Endres JL, Mann EE, Sadykov MR, Horswill AR, Rice KC, Fey PD, Bayles KW. Use of microfluidic technology to analyze gene expression during *Staphylococcus aureus* biofilm formation reveals distinct physiological niches. Appl Environ Microbiol 2013; 79:3413-24; PMID:23524683; http://dx.doi.org/10.1128/AEM.00395-13 23524683 PMC3648040

[143] Sun F, Ji Q, Jones MB, Deng X, Liang H, Frank B, Telser J, Peterson SN, Bae T, He C. AirSR, a [2Fe-2S] cluster-containing two-component system, mediates global oxygen sensing and redox signaling in *Staphylococcus aureus*. J Am Chem Soc 2012; 134:305-14; PMID:22122613; http://dx.doi.org/10.1021/ja207 1835 22122613 PMC3257388

[144] Yan M, Hall JW, Yang J, Ji Y. The essential yhcSR two-component signal transduction system directly regulates the lac and opuCABCD operons of *Staphylococcus aureus*. PLoS One 2012; 7:e50608; PMID:23226327; http://dx.doi.org/10.1371/journal.pone.0050608 23226327 PMC3511567

[145] Gusarov I, Starodubtseva M, Wang ZQ, McQuade L, Lippard SJ, Stuehr DJ, Nudler E. Bacterial nitric-oxide synthases operate without a dedicated redox partner. J Biol Chem 2008; 283:13140-7; PMID:18316370; http://dx.doi.org/10.1074/jbc.M710178200 18316370 PMC2442334

[146] Gusarov I, Shatalin K, Starodubtseva M, Nudler E. Endogenous nitric oxide protects bacteria against a wide spectrum of antibiotics. Science 2009; 325:1380-4; PMID:19745150; http://dx.doi.org/10.1126/science.1175439 19745150 PMC2929644

[147] Gusarov I, Nudler E. NO-mediated cytoprotection: instant adaptation to oxidative stress in bacteria. Proc Natl Acad Sci U S A 2005; 102:13855-60; PMID:16172391; http://dx.doi.org/10.1073/pnas.0504307102 16172391 PMC1236549

[148] Shatalin K, Gusarov I, Avetissova E, Shatalina Y, McQuade LE, Lippard SJ, Nudler E. *Bacillus anthracis*-derived nitric oxide is essential for pathogen virulence and survival in macrophages. Proc Natl Acad Sci U S A 2008; 105:1009-13; PMID:18215992; http://dx.doi.org/10.1073/pnas.0710950105 18215992 PMC2242674

[149] Kers JA, Wach MJ, Krasnoff SB, Widom J, Cameron KD, Bukhalid RA, Gibson DM, Crane BR, Loria R. Nitration of a peptide phytotoxin by bacterial nitric oxide synthase. Nature 2004; 429:79-82; PMID:15129284; http://dx.doi.org/10.1038/nature02504 15129284

[150] van Sorge NM, Beasley FC, Gusarov I, Gonzalez DJ, von Köckritz-Blickwede M, Anik S, Borkowski AW, Dorrestein PC, Nudler E, Nizet V. Methicillin-resistant *Staphylococcus aureus* bacterial nitric-oxide synthase affects antibiotic sensitivity and skin abscess development. J Biol Chem 2013; 288:6417-26; PMID:23322784; http://dx.doi.org/10.1074/jbc.M112.448738 23322784 PMC3585076

[151] Vaish M, Singh VK. Antioxidant functions of nitric oxide synthase in a methicillin sensitive *Staphylococcus aureus*. Int J Microbiol 2013; 2013:312146; PMID:23690783; http://dx.doi.org/10.1155/2013/312146 23690783 PMC3638668

[152] Richardson AR, Dunman PM, Fang FC. The nitrosative stress response of *Staphylococcus aureus* is required for resistance to innate immunity. Mol Microbiol 2006; 61:927-39; PMID:16859493; http://dx.doi.org/10.1111/j.1365-2958.2006.05290.x 16859493

[153] Richardson AR, Libby SJ, Fang FC. A nitric oxide-inducible lactate dehydrogenase enables *Staphylococcus aureus* to resist innate immunity. Science 2008; 319:1672-6; PMID:18356528; http://dx.doi.org/10.1126/science.1155207 18356528

[154] Gonçalves VL, Nobre LS, Vicente JB, Teixeira M, Saraiva LM. Flavohemoglobin requires microaerophilic conditions for nitrosative protection of *Staphylococcus aureus*. FEBS Lett 2006; 580:1817-21; PMID:16516202; http://dx.doi.org/10.1016/j.febslet.2006.02.039 16516202

[155] World Health Organisation (2011) Global Tuberculosis Control. WHO Press, Geneva, WHO/HTM/TB/2011.16.

[156] O’Garra A, Redford PS, McNab FW, Bloom CI, Wilkinson RJ, Berry MP. The immune response in tuberculosis. Annu Rev Immunol 2013; 31:475-527; PMID:23516984; http://dx.doi.org/10.1146/annurev-immunol-032712-095939 23516984

[157] Russell DG. *Mycobacterium tuberculosis*: here today, and here tomorrow. Nat Rev Mol Cell Biol 2001; 2:569-77; PMID:11483990; http://dx.doi.org/10.1038/35085034 11483990

[158] Russell DG. Who puts the tubercle in tuberculosis? Nat Rev Microbiol 2007; 5:39-47; PMID:17160001; http://dx.doi.org/10.1038/nrmicro1538 17160001

[159] Russell DG, Barry CE 3rd, Flynn JL. Tuberculosis: what we don't know can, and does, hurt us. Science 2010; 328:852-6; PMID:20466922; http://dx.doi.org/10.1126/science.1184784 20466922 PMC2872107

[160] Via LE, Lin PL, Ray SM, Carrillo J, Allen SS, Eum SY, Taylor K, Klein E, Manjunatha U, Gonzales J, et al. Tuberculous granulomas are hypoxic in guinea pigs, rabbits, and nonhuman primates. Infect Immun 2008; 76:2333-40; PMID:18347040; http://dx.doi.org/10.1128/IAI.01515-07 18347040 PMC2423064

[161] Wayne LG, Sohaskey CD. Nonreplicating persistence of *mycobacterium tuberculosis*. Annu Rev Microbiol 2001; 55:139-63; PMID:11544352; http://dx.doi.org/10.1146/annurev.micro.55.1.139 11544352

[162] Voskuil MI, Schnappinger D, Visconti KC, Harrell MI, Dolganov GM, Sherman DR, Schoolnik GK. Inhibition of respiration by nitric oxide induces a *Mycobacterium tuberculosis* dormancy program. J Exp Med 2003; 198:705-13; PMID:12953092; http://dx.doi.org/10.1084/jem.20030205 12953092 PMC2194188

[163] Sardiwal S, Kendall SL, Movahedzadeh F, Rison SC, Stoker NG, Djordjevic S. A GAF domain in the hypoxia/NO-inducible *Mycobacterium tuberculosis* DosS protein binds haem. J Mol Biol 2005; 353:929-36; PMID:16213520; http://dx.doi.org/10.1016/j.jmb.2005.09.011 16213520

[164] Sousa EH, Tuckerman JR, Gonzalez G, Gilles-Gonzalez MA. DosT and DevS are oxygen-switched kinases in *Mycobacterium tuberculosis*. Protein Sci 2007; 16:1708-19; PMID:17600145; http://dx.doi.org/10.1110/ps.072897707 17600145 PMC2203369

[165] Kumar A, Toledo JC, Patel RP, Lancaster JR Jr., Steyn AJ. *Mycobacterium tuberculosis* DosS is a redox sensor and DosT is a hypoxia sensor. Proc Natl Acad Sci U S A 2007; 104:11568-73; PMID:17609369; http://dx.doi.org/10.1073/pnas.0705054104 17609369 PMC1906723

[166] Ioanoviciu A, Meharenna YT, Poulos TL, Ortiz de Montellano PR. DevS oxy complex stability identifies this heme protein as a gas sensor in *Mycobacterium tuberculosis* dormancy. Biochemistry 2009; 48:5839-48; PMID:19463006; http://dx.doi.org/10.1021/bi802309y 19463006 PMC2756985

[167] Sivaramakrishnan S, Ortiz de Montellano PR The DosS-DosT/DosR mycobacterial sensor system. Biosensors 2013; 3:259-82; http://dx.doi.org/10.3390/bios3030259 25002970 PMC4082495

[168] Yukl ET, Ioanoviciu A, Sivaramakrishnan S, Nakano MM, Ortiz de Montellano PR, Moënne-Loccoz P. Nitric oxide dioxygenation reaction in DevS and the initial response to nitric oxide in *Mycobacterium tuberculosis*. Biochemistry 2011; 50:1023-8; PMID:21250657; http://dx.doi.org/10.1021/bi1015315 21250657 PMC3079480

[169] Honaker RW, Leistikow RL, Bartek IL, Voskuil MI. Unique roles of DosT and DosS in DosR regulon induction and *Mycobacterium tuberculosis* dormancy. Infect Immun 2009; 77:3258-63; PMID:19487478; http://dx.doi.org/10.1128/IAI.01449-08 19487478 PMC2715697

[170] Vos MH, Bouzhir-Sima L, Lambry JC, Luo H, Eaton-Rye JJ, Ioanoviciu A, Ortiz de Montellano PR, Liebl U Ultrafast ligand dynamics in the heme-based GAF sensor domains of the histidine kinases DosS and DosT from *Mycobacterium tuberculosis*. Biochemistry 2012; 51:159-66; PMID:2067548022142262; http://dx.doi.org/10.1021/bi201467c 22142262 PMC3254832

[171] Kim MJ, Park KJ, Ko IJ, Kim YM, Oh JI. Different roles of DosS and DosT in the hypoxic adaptation of Mycobacteria. J Bacteriol 2010; 192:4868-75; PMID:20675480; http://dx.doi.org/10.1128/JB.00550-10 20675480 PMC2944544

[172] Boon C, Dick T. *Mycobacterium bovis* BCG response regulator essential for hypoxic dormancy. J Bacteriol 2002; 184:6760-7; PMID:12446625; http://dx.doi.org/10.1128/JB.184.24.6760-6767.2002 12446625 PMC135468

[173] Park HD, Guinn KM, Harrell MI, Liao R, Voskuil MI, Tompa M, Schoolnik GK, Sherman DR. Rv3133c/*dosR* is a transcription factor that mediates the hypoxic response of *Mycobacterium tuberculosis*. Mol Microbiol 2003; 48:833-43; PMID:12694625; http://dx.doi.org/10.1046/j.1365-2958.2003.03474.x 12694625 PMC1992516

[174] Voskuil MI, Visconti KC, Schoolnik GK. *Mycobacterium tuberculosis* gene expression during adaptation to stationary phase and low-oxygen dormancy. Tuberculosis (Edinb) 2004; 84:218-27; PMID:15207491; http://dx.doi.org/10.1016/j.tube.2004.02.003 15207491

[175] Gengenbacher M, Kaufmann SH. *Mycobacterium tuberculosis*: success through dormancy. FEMS Microbiol Rev 2012; 36:514-32; PMID:22320122; http://dx.doi.org/10.1111/j.1574-6976.2012.00331.x 22320122 PMC3319523

[176] Leistikow RL, Morton RA, Bartek IL, Frimpong I, Wagner K, Voskuil MI. *The Mycobacterium tuberculosis* DosR regulon assists in metabolic homeostasis and enables rapid recovery from nonrespiring dormancy. J Bacteriol 2010; 192:1662-70; PMID:20023019; http://dx.doi.org/10.1128/JB.00926-09 20023019 PMC2832541

[177] Rustad TR, Sherrid AM, Minch KJ, Sherman DR. Hypoxia: a window into M*ycobacterium tuberculosis* latency. Cell Microbiol 2009; 11:1151-9; PMID:19388905; http://dx.doi.org/10.1111/j.1462-5822.2009.01325.x 19388905

[178] den Hengst CD, Buttner MJ. Redox control in actinobacteria. Biochim Biophys Acta 2008; 1780:1201-16; PMID:18252205; http://dx.doi.org/10.1016/j.bbagen.2008.01.008 18252205

[179] Soliveri JA, Gomez J, Bishai WR, Chater KF. Multiple paralogous genes related to the *Streptomyces coelicolor* developmental regulatory gene *whiB* are present in *Streptomyces* and other actinomycetes. Microbiology 2000; 146:333-43; PMID:10708372 10708372 10.1099/00221287-146-2-333

[180] Davis NK, Chater KF. The *Streptomyces coelicolor whiB* gene encodes a small transcription factor-like protein dispensable for growth but essential for sporulation. Mol Gen Genet 1992; 232:351-8; PMID:1316997; http://dx.doi.org/10.1007/BF00266237 1316997

[181] Alam MS, Garg SK, Agrawal P. Molecular function of WhiB4/Rv3681c of *Mycobacterium tuberculosis* H37Rv: a [4Fe-4S] cluster co-ordinating protein disulphide reductase. Mol Microbiol 2007; 63:1414-31; PMID:17302817; http://dx.doi.org/10.1111/j.1365-2958.2007.05589.x 17302817

[182] Alam MS, Garg SK, Agrawal P. Studies on structural and functional divergence among seven WhiB proteins of *Mycobacterium tuberculosis* H37Rv. FEBS J 2009; 276:76-93; PMID:19016840; http://dx.doi.org/10.1111/j.1742-4658.2008.06755.x 19016840

[183] Jakimowicz P, Cheesman MR, Bishai WR, Chater KF, Thomson AJ, Buttner MJ. Evidence that the *Streptomyces* developmental protein WhiD, a member of the WhiB family, binds a [4Fe-4S] cluster. J Biol Chem 2005; 280:8309-15; PMID:15615709; http://dx.doi.org/10.1074/jbc.M412622200 15615709

[184] Rybniker J, Nowag A, van Gumpel E, Nissen N, Robinson N, Plum G, Hartmann P. Insights into the function of the WhiB-like protein of mycobacteriophage TM4–a transcriptional inhibitor of WhiB2. Mol Microbiol 2010; 77:642-57; PMID:20545868; http://dx.doi.org/10.1111/j.1365-2958.2010.07235.x 20545868

[185] Singh A, Guidry L, Narasimhulu KV, Mai D, Trombley J, Redding KE, Giles GI, Lancaster JR Jr., Steyn AJ. *Mycobacterium tuberculosis* WhiB3 responds to O_2_ and nitric oxide via its [4Fe-4S] cluster and is essential for nutrient starvation survival. Proc Natl Acad Sci U S A 2007; 104:11562-7; PMID:17609386; http://dx.doi.org/10.1073/pnas.0700490104 17609386 PMC1906726

[186] Smith LJ, Stapleton MR, Fullstone GJ, Crack JC, Thomson AJ, Le Brun NE, Hunt DM, Harvey E, Adinolfi S, Buxton RS, et al. *Mycobacterium tuberculosis* WhiB1 is an essential DNA-binding protein with a nitric oxide-sensitive iron-sulfur cluster. Biochem J 2010; 432:417-27; PMID:20929442; http://dx.doi.org/10.1042/BJ20101440 20929442 PMC2992795

[187] Smith LJ, Stapleton MR, Buxton RS, Green J. Structure-function relationships of the *Mycobacterium tuberculosis* transcription factor WhiB1. PLoS One 2012; 7:e40407; PMID:22792304; http://dx.doi.org/10.1371/journal.pone.0040407 22792304 PMC3390391

[188] Crack JC, Smith LJ, Stapleton MR, Peck J, Watmough NJ, Buttner MJ, Buxton RS, Green J, Oganesyan VS, Thomson AJ, et al. Mechanistic insight into the nitrosylation of the [4Fe-4S] cluster of WhiB-like proteins. J Am Chem Soc 2011; 133:1112-21; PMID:21182249; http://dx.doi.org/10.1021/ja109581t 21182249 PMC3117330

[189] Stapleton MR, Smith LJ, Hunt DM, Buxton RS, Green J. *Mycobacterium tuberculosis* WhiB1 represses transcription of the essential chaperonin GroEL2. Tuberculosis (Edinb) 2012; 92:328-32; PMID:22464736; http://dx.doi.org/10.1016/j.tube.2012.03.001 22464736 PMC3430963

[190] Guo M, Feng H, Zhang J, Wang W, Wang Y, Li Y, Gao C, Chen H, Feng Y, He ZG. Dissecting transcription regulatory pathways through a new bacterial one-hybrid reporter system. Genome Res 2009; 19:1301-8; PMID:19228590; http://dx.doi.org/10.1101/gr.086595.108 19228590 PMC2704442

[191] Saini V, Farhana A, Steyn AJ. *Mycobacterium tuberculosis* WhiB3: a novel iron-sulfur cluster protein that regulates redox homeostasis and virulence. Antioxid Redox Signal 2012; 16:687-97; PMID:22010944; http://dx.doi.org/10.1089/ars.2011.4341 22010944 PMC3277930

[192] Singh A, Crossman DK, Mai D, Guidry L, Voskuil MI, Renfrow MB, Steyn AJ. *Mycobacterium tuberculosis* WhiB3 maintains redox homeostasis by regulating virulence lipid anabolism to modulate macrophage response. PLoS Pathog 2009; 5:e1000545; PMID:19680450; http://dx.doi.org/10.1371/journal.ppat.1000545 19680450 PMC2718811

[193] Steyn AJ, Collins DM, Hondalus MK, Jacobs WR Jr., Kawakami RP, Bloom BR. *Mycobacterium tuberculosis* WhiB3 interacts with RpoV to affect host survival but is dispensable for *in vivo* growth. Proc Natl Acad Sci U S A 2002; 99:3147-52; PMID:11880648; http://dx.doi.org/10.1073/pnas.052705399 11880648 PMC122487

[194] Banaiee N, Jacobs WR Jr., Ernst JD. Regulation of *Mycobacterium tuberculosis whiB3* in the mouse lung and macrophages. Infect Immun 2006; 74:6449-57; PMID:16923787; http://dx.doi.org/10.1128/IAI.00190-06 16923787 PMC1695489

[195] Geiman DE, Raghunand TR, Agarwal N, Bishai WR. Differential gene expression in response to exposure to antimycobacterial agents and other stress conditions among seven *Mycobacterium tuberculosis whiB*-like genes. Antimicrob Agents Chemother 2006; 50:2836-41; PMID:16870781; http://dx.doi.org/10.1128/AAC.00295-06 16870781 PMC1538666

[196] Larsson C, Luna B, Ammerman NC, Maiga M, Agarwal N, Bishai WR. Gene expression of *Mycobacterium tuberculosis* putative transcription factors *whiB1–7* in redox environments. PLoS One 2012; 7:e37516; PMID:22829866; http://dx.doi.org/10.1371/journal.pone.0037516 22829866 PMC3400605

